# Epidemic Waves and Exact Solutions of a Sequence of Nonlinear Differential Equations Connected to the SIR Model of Epidemics

**DOI:** 10.3390/e25030438

**Published:** 2023-03-01

**Authors:** Nikolay K. Vitanov, Kaloyan N. Vitanov

**Affiliations:** 1 Institute of Mechanics, Bulgarian Academy of Sciences, Acad. G. Bonchev Str., Bl. 4, 1113 Sofia, Bulgaria; 2 Climate, Atmosphere and Water Research Institute, Bulgarian Academy of Sciences, Blvd. Tzarigradsko Chaussee 66, 1784 Sofia, Bulgaria

**Keywords:** SIR model of epidemics, nonlinear differential equations, exact solutions, Simple Equations Method (SEsM), epidemic waves, COVID-19, real data for epidemic spreading of COVID-19

## Abstract

The SIR model of epidemic spreading can be reduced to a nonlinear differential equation with an exponential nonlinearity. This differential equation can be approximated by a sequence of nonlinear differential equations with polynomial nonlinearities. The equations from the obtained sequence are treated by the Simple Equations Method (SEsM). This allows us to obtain exact solutions to some of these equations. We discuss several of these solutions. Some (but not all) of the obtained exact solutions can be used for the description of the evolution of epidemic waves. We discuss this connection. In addition, we use two of the obtained solutions to study the evolution of two of the COVID-19 epidemic waves in Bulgaria by a comparison of the solutions with the available data for the infected individuals.

## 1. Introduction

There are many complex systems of various scales around us. Examples range from atomic chains and lattices to systems of animals, humans and groups of humans, for example, research groups and social networks, economic systems, etc. [1,2,3,4,5,6,7]. These complex systems are usually nonlinear [8,9,10], and this complicates the study. One has to use the methodology of nonlinear time series analysis, and many of the corresponding models are based on differential or difference equations [11,12,13,14]. Then, one has to obtain solutions to these nonlinear equations. Usually, numerical methods are used to obtain the needed solutions. However, it is very useful if one can obtain exact analytical solutions to the model equations. In this case, one can easily study the relationships among the parameters of the complex system of interest. In addition, the exact solutions can be used as test solutions for checking the correctness of the work of the computer programs that have to supply numerical solutions to the corresponding systems of equations. In this article, we consider a nonlinear model of the evolution of epidemic waves (the SIR model of epidemics) and discuss a methodology for obtaining analytical solutions connected to this model. The methodology is based on the reduction of the model to a sequence of nonlinear differential equations. Several exact solutions to these nonlinear differential equations are obtained. Some of the solutions can be used for descriptions of epidemic waves.

Because of its importance, there is a large amount of research on the methodology for obtaining exact solutions to nonlinear differential equations. In the beginning, one tried to remove the nonlinearity of the studied equation by an appropriate transformation, such as the Hopf–Cole transformation [15,16]. Another transformation connected the nonlinear Korteweg–de Vries equation to the linear Schrödinger equation, and this led to the method of inverse scattering transform [17]. Other suitable transformations can be supplied by the truncated Painleve expansions [18,19,20,21,22]. Such a study led Kudryashov [23] to the formulation of the Method of Simplest Equation (MSE). The method is based on the determination of singularity order *n* of the solved NPDE. Then, a particular solution to this equation is searched as power series of solutions to a simpler equation called the simplest equation. For further results on this methodology and its applications, see [24,25,26,27,28].

Below we will use a methodology called SEsM (Simple Equations Method) [29,30,31,32,33]. Some specific cases of this methodology can be seen in our articles written approximately 30 years ago [34,35]. More than a decade ago [36], we used the ODE of Bernoulli as the simplest equation in the first version of the method: Modified Method of Simplest Equation (MMSE). We applied the methodology to population dynamics and ecology [37]. The MMSE [38] uses the concept of balanced equations for the fixation of the simplest equation. After this, the searched solution was constructed as a truncated power series of the solution to the simplest equation. This methodology leads to equivalent results with respect to the Method of Simplest Equation of Kudryashov.

We used MMSE actively till 2018 [39,40]. Our efforts have been constantly directed to extend the capacity of the methodology. The current version of the methodology (SEsM) can use more than one simple equation. SEsM based on two simple equations was used in [41]. The first discussion of SEsM was in [29]. Further discussion on SEsM can be seen in [33]. Applications of specific cases of SEsM can be seen in [42,43].

Below we apply SEsM to a system of nonlinear differential equations connected to an epidemic model: the SIR model of the spreading of epidemics in a population. There exist many models for the spread of epidemics. One of the most basic of these models is the SIR model for describing the temporal dynamics of an infectious disease in a population. The model compartmentalizes people into one of three categories: (i) susceptible to the disease; (ii) those who are currently infectious, and (iii) those who have recovered (with immunity). The SIR model is a set of equations that describes the number of people in each compartment at every point in time. A large amount of literature is devoted to this topic (for several examples, see [44,45,46,47,48,49,50,51,52,53,54,55,56,57,58,59,60,61,62]). Epidemic models can also be applied for the description of other processes, such as the spread of ideas (for overviews, see [3,63]). We also note the use of epidemic models for the study of COVID-19 spreading [64,65,66,67,68,69,70,71,72,73,74,75,76,77,78], as well as the numerical methods for obtaining solutions to such models [79,80].

We note the implicit solutions to the SIR model obtained by means of the Lambert function [81] and in [82]. Note also that the SIR model does not pass the Painleve test [81]. The solutions obtained below are explicit ones, and they are constructed by the use of elementary functions.

The text is organized as follows. In Section 2, we describe the methodology of SEsM. In Section 3, we obtain several exact solutions to the chain of nonlinear differential equations connected to the SIR model of epidemic spreading. Some of these solutions are appropriate to model epidemic waves. We discuss them in Section 4. In Section 5, we study the influence of the parameters of the SIR model on the shape of the epidemic waves described by the obtained solutions. In the same section, we use the obtained solutions to study two of the COVID-19 epidemic waves in Bulgaria. Several concluding remarks are summarized in Section 6.

## 2. Simple Equations Method (SEsM)

In general, the SEsM is constructed for obtaining exact and approximate solutions to systems of nonlinear differential equations. We will introduce a notation for the classification of the different cases of SEsM. In general, we have to solve *n* nonlinear differential equations. The main idea of SEsM is to obtain a solution on the basis of known solutions to *m* simpler differential equations. We denote this version of SEsM by SEsM(n,m). The most used version of SEsM up to now is this one for n=1. In this case, one has to solve one complicated nonlinear differential equation by means of the known solutions to *m* simpler differential equations. This will be denoted as SEsM(1,m). The most used specific version of SEsM(1,m) is the version SEsM(1,1). In this case, we solve one complicated nonlinear differential equation by means of the known solutions to one simpler differential equation. SEsM(1,1) is called also the Modified Method of Simplest Equation, and there are many applications of this specific case of SEsM [83,84,85].

The main idea of SEsM is as follows. We have to solve a system of nonlinear differential equations:

(1) $$D_{i}\left[u_{i1}\left(x,\ldots ,t\right),\ldots ,u_{in}\left(x,\ldots ,t\right)\right]=0,\;\;\;i=1,2,\ldots ,n.$$

$D_{i}\left[u_{i1}\left(x,\ldots ,t\right),\ldots ,u_{in}\left(x,\ldots ,t\right),\ldots \right]$ depends on the functions $u_{i1}\left(x,\ldots ,t\right),\ldots ,u_{in}\left(x,\ldots ,t\right)$ and some of their derivatives. The functions $u_{ik}$ can depend on several spatial coordinates. We have to transform (1) to

(2) $$\sum_{i=1}^{n}a_{ij}\left(\ldots \right)E_{ij}=0,j=1,2,\ldots ,p_{i}.$$

This happens by the representation of $u_{ik}$ as composite functions of known analytical solutions to simpler equations. Above, $E_{ij}$ are functions of the independent spatial variables and of time. The quantities $a_{ij}$ are very important for SEsM. $a_{ij}$ are relationships among the parameters of the Equation (1), parameters of the solutions and the parameters of the solutions to the simpler equations. $p_{i}$ is a parameter that is characteristic of the *i*-th equation from (1). It is important that the relationships $a_{ij}$ contain only parameters, whereas the spatial coordinates and the time are concentrated on the functions $E_{ij}$. If we manage to reduce the Equation (1) to the form (2), then we can set

(3) $$a_{ij}\left(\ldots \right)=0.$$

Thus, we obtain a system of nonlinear algebraic equations. This system contains relationships among the parameters of (1), parameters of the used simpler equations and the parameters of the solution constructed by the simpler equations. Each nontrivial solution to (3) leads to a solution to the system (1).

SEsM has four steps. The first step of SEsM is connected to the possibility of transformations of the nonlinearities of the Equation (1). The solved complicated differential equations contain nonlinear combinations of the unknown functions and their derivatives. The experience shows that polynomial nonlinearity is the most treatable kind of nonlinearity in a nonlinear differential equation. Thus, if the nonlinearities in (1) are polynomial ones, then one does not need a transformation to convert these nonlinearities. However, if the nonlinearities are not polynomial ones, then one can use the transformation order to convert the nonlinearities to more treatable kinds of nonlinearities (and eventually to polynomial nonlinearities). Thus, one applies the transformations

(4) $$u_{ik}\left(x,...,t\right)=T_{ik}\left[F_{ikl}\left(x,\ldots ,t\right),G_{ikl}\left(x,\ldots ,t\right),\ldots \right].$$

In (4), $T_{ik}\left(F_{ikl},G_{ikl},\ldots \right)$ is a composite function of other functions $F_{ikl},G_{ikl},\ldots$. These other functions can be functions of several spatial variables and time. The transformations have two goals. First of all, and if it is possible, the transformations may remove the nonlinearity of the solved Equation (1). One example of this is the Hopf–Cole transformation. This transformation reduces the nonlinear Burgers equation to the linear heat equation [15,16]. However, removing the nonlinearity of an equation by means of a transformation is rarely achieved. Usually, the transformations $T_{ik}$ intend to transform the nonlinearity of the solved equations to a more treatable kind of nonlinearity, such as polynomial nonlinearity. For the specific case of SEsM(1,1), examples of the transformation *T* are: $u\left(x,t\right)=4\tanh^{-1}\left[F\left(x,t\right)\right]$ for the Poisson–Boltzmann equation, and $u\left(x,t\right)=4\tan^{-1}\left[F\left(x,t\right)\right]$ for the Sine–Gordon equation [34,35]. The Painleve expansion is another appropriate transformation. Finally, u(x,...,t)=F(x,...,t) (no transformation) is a possibility for certain classes of nonlinear differential equations. The application of (4) to (1) may lead to treatable nonlinear differential equations for $F_{ikl},G_{i,kl},\ldots$. The transformations $T_{ik}$ may remain unfixed at the first step of SEsM. Then, the functions $T_{ik}$ remain unknown, and we have to determine them at some of the following three steps of the methodology.

Step 2 of SEsM is connected to the construction of the solutions to the transformed Equation (1). The transformed equations contain the unknown functions and their derivatives. The basic idea of SEsM is to search for solutions to these equations in the form of composite functions containing solutions to simpler differential equations. The implementation of this idea leads to the necessity of working with derivatives of composite functions. These derivatives have to be expressed by the solutions to simpler equations and derivatives of these solutions. Because of this, one has to use the Faa di Bruno formula for the derivatives of the composite functions. In such a way, the solved equations are converted to relationships that contain functions that are solutions to more simple equations. At this point of the application of SEsM, the composite functions and the solutions to the more simple equation are still not fixed. We can fix the forms of the composite function in Step 2 of SEsM. However, in general, it is not necessary to do this at this step. One example of a fixation of the composite function for the case of SEsM(1,1) is

(5) $$\begin{array}{c} F=\alpha +\sum_{i_{1}=1}^{N}\beta_{i_{1}}f_{i_{1}}+\sum_{i_{1}=1}^{N}\sum_{i_{2}=1}^{N}\gamma_{i_{1},i_{2}}f_{i_{1}}f_{i_{2}}+\sum_{i_{1}=1}^{N}\cdots \sum_{i_{N}=1}^{N}\sigma_{i_{1},\ldots ,i_{N}}f_{i_{1}}\ldots f_{i_{N}}. \end{array}$$

where $\alpha ,\beta_{i_{1}},\gamma_{i_{1},i_{2}},\sigma_{i_{1},\ldots ,i_{N}}\ldots$ are parameters, and $f_{i_{k}}$ are functions that are solutions to more simple equations. The relationship used by Hirota [86] is a specific case of (5).

Step 3 of SEsM is usually the most important step in the application of the methodology. Here, we determine the form of the more simple equations of which the solutions will be used for solutions to the system of solved nonlinear differential equations. The simple equations, as well as the composite functions constructed by their solutions, are chosen in such a way that the solved differential equations are transformed into a system of relationships (1). One has to ensure that the relationships for $a_{ij}$ contain more than one term. Because of this, additional relationships among the parameters participating in the relationships for $a_{ij}$ may occur. These additional relationships are called balance equations.

At step 4 of SEsM, we use (3). This leads to a system of nonlinear algebraic relationships among the parameters of the Equation (1), the parameters of the composite functions, and the parameters of the solutions to the simpler equations. Any nontrivial solution to (3) leads to a solution to the system of the solved nonlinear equations.

For a specific case of applications of SEsM, see [29,30,31,32,33,36,37,38,39,40,41].

## 3. SEsM and Exact Analytical Solutions for a Chain of Equations Connected to the SIR Model of Epidemics

Below we use SEsM to obtain exact solutions to a chain of nonlinear differential equations connected to a specific nonlinear differential equation. This equation will be obtained on the basis of the SIR model from the epidemiology. Some of the obtained solutions can be used to the description of epidemic waves caused by different diseases (COVID-19 inclusive). Other obtained solutions will not be appropriate for this purpose.

The basic nonlinear differential equation is obtained by the use of the classic idea of Kermack and McKendrick [87] for the transformation of the SIR model with constant coefficients to a single nonlinear differential equation. We consider an epidemic in a population. The population is divided into three groups: susceptible individuals—*S*; infected individuals—*I*; recovered individuals—*R*. The model equations for the time change in the numbers of individuals from the above three groups are:

(6) $$\begin{array}{ccc} \frac{dS}{dt} & = & -\frac{\tau }{N}SI\\ \frac{dI}{dt} & = & \frac{\tau }{N}SI-\rho I\\ \frac{dR}{dt} & = & \rho I. \end{array}$$

In (6), τ is the transmission rate, and ρ is the recovery rate. These rates are assumed to be constants. From (6), we have the relationship

(7) N=S+I+R.

*N* is the total population, which is assumed to be constant. The model (6) can be reduced to a single equation for *R*, as follows. From the last equation of (6), we have

(8) $$I=\frac{1}{\rho }\frac{dR}{dt}.$$

The substitution of (8) in the first equation of (6) leads to the relationship

(9) $$S=S\left(0\right)\exp\left\{-\frac{\tau }{\rho N}\left[R-R\left(0\right)\right]\right\}.$$

Here, S(0) and R(0) are the numbers of susceptible individuals and those recovered at time t=0. The substitution of (7) and (9) in the last equation of (6) leads to the differential equation for *R*

(10) $$\frac{dR}{dt}=\rho \left\{N-R-S\left(0\right)\exp\left[-\frac{\tau }{\rho N}\left(R-R\left(0\right)\right)\right]\right\}$$

Below, we assume R(0)=0 (no recovered individuals at t=0). Let us consider the ratio $\frac{\tau R}{\rho N}$. We assume that $\frac{\tau R}{\rho N}<<1$. This can be realized, for example, when τ>ρ and R<<N. This means that we have an epidemic wave that affects a small amount of the population, and the number of recovered people remains small with respect to the number of the entire population. In this case, $\exp\left[-\frac{\tau }{\rho N}R\right]$ can be represented as a Taylor series

(11) $$\exp\left[-\frac{\tau }{\rho N}R\right]=\sum_{j=0}^{M}{\left(-\frac{\tau }{\rho N}R\right)}^{j}.$$

*M* has infinite value in the full Taylor series, but we can truncate it at M=2, M=3,..., if $-\frac{\tau }{\rho N}R$ is small enough. From (10), we obtain

(12) $$\frac{dR}{dt}=\rho \left\{N-R-S\left(0\right)\sum_{j=0}^{M}{\left(-\frac{\tau }{\rho N}R\right)}^{j}\right\},\;\;\;M=2,3,\ldots$$

We set

(13) $$\alpha_{0}=\rho \left[N-S\left(0\right)\right];\;\;\;\alpha_{1}=\frac{\tau S(0)}{N}-\rho ;\;\;\;\alpha_{j}=-{\left(-1\right)}^{j}\frac{\tau^{j}S\left(0\right)}{\rho^{j-1}N^{j}},\;\;j=2,3,\ldots$$

Then (12) becomes

(14) $$\frac{dR}{dt}=\sum_{j=0}^{M}\alpha_{j}R^{j}$$

The chains of Equation (12) and (14) are connected to the orders of approximation of (10), which is the equation for the time evolution of the recovering individuals for an epidemic wave within the scope of the SIR model.

In (14), the independent variable is the time *t*. In principle, the independent variable can also be a spatial coordinate or a combination of spatial variables and time. In order to discuss this (more general) case below, we use an independent variable denoted as *x*. This variable can be a spatial variable, time, or some combination of spatial variables and time. Thus, we apply SEsM to the equation below

(15) $$\frac{dR}{dx}=\sum_{j=0}^{M}\alpha_{j}R^{j},$$

and we use the differential equations of Bernoulli and Riccati as simple equations. Our plan is as follows. First, we describe exact analytical solutions to the equations of the chain (14). Then, we adapt these solutions for the specific case of epidemics described by the chain of Equation (12).

First of all, we use the equation of Bernoulli

(16) $$\frac{dy}{dx}=py+qy^{m},\;\;\;m=2,3,\ldots ,$$

as a simple equation within the scope of the SEsM methodology. By means of the transformation, $y=u^{1/(1-m)}$ (16) is reduced to a linear differential equation. This leads to the solution to the equation of Bernoulli as follows

(17) $$y\left(x\right)={\left\{\frac{p}{-q+Cp\exp[-(m-1)px]}\right\}}^{\frac{1}{m-1}}.$$

In (17), *C* is a constant of integration.

We skip Step 1 of SEsM. No transformation is needed because the kind of nonlinearity of (15) is a polynomial one. In Step 2 of SEsM, we prescribe the composite function R(y) to be of the kind

(18) $$R\left(y\right)=\sum_{l=0}^{L}\beta_{l}y^{l},$$

where y(x) is the solution to the equation of Bernoulli and R(y) is the solution to (15). At Step 3 of SEsM, we have to obtain the balance equation. The presence of (16) and (18) fixes the balance equation of (15) to

(19) m=1+L(M−1).

Thus, a specific solution to (15) has the form

(20) $$R\left(x\right)=\sum_{l=0}^{L}\beta_{l}{\left\{\frac{p}{-q+Cp\exp\{-[L(M-1)]px\}}\right\}}^{\frac{l}{L(M-1)}}.$$

The parameters $\beta_{l}$, *p*, *q* and *C* are fixed by the solution to the system of nonlinear algebraic equations at Step 4 of SEsM.

There is a specific case where we can obtain the general solution of (15). This case is M=2. Here, (15) becomes

(21) $$\frac{dR}{dx}=\alpha_{2}R^{2}+\alpha_{1}R+\alpha_{0}.$$

(21) is an equation of the Riccati kind. For this equation, we know the specific solution

(22) $$R\left(x\right)=-\frac{\alpha_{1}}{2\alpha_{2}}-\frac{\theta }{2\alpha_{2}}\tanh\left[\frac{\theta (x+C)}{2}\right],$$

where $\theta^{2}=\alpha_{1}^{2}-4\alpha_{0}\alpha_{2}>0$, and *C* is a constant of integration. On the basis of the specific solution (22) of (21), we can write the general solution to (21) as $R=-\frac{\alpha_{1}}{2\alpha_{2}}-\frac{\theta }{2\alpha_{2}}\tanh\left[\frac{\theta (x+C)}{2}\right]+\frac{D}{v}$ where *D* is a constant, and v(t) is the solution to the linear differential equation

(23) $$\frac{dv}{dx}-\theta \tanh\left[\frac{\theta (x+C)}{2}\right]v=-\alpha_{2}D$$

The solution to (23) is

(24) $$v=\cosh^{2}\left[\frac{\theta (x+C}{2}\right]\left\{E-\frac{2\alpha_{2}D}{\theta }\tanh\left[\frac{\theta (x+C}{2}\right]\right\},$$

where *E* is a constant of integration. Then, the general solution to Equation (21) is

(25) $$R\left(x\right)=-\frac{\alpha_{1}}{2\alpha_{2}}-\frac{\theta }{2\alpha_{2}}\tanh\left[\frac{\theta (x+C)}{2}\right]+\frac{D}{\cosh^{2}\left[\frac{\theta (x+C)}{2}\right]\left\{E-\frac{2\alpha_{2}D}{\theta }\tanh\left[\frac{\theta (x+C)}{2}\right]\right\}}.$$

Let us now obtain the form of several solutions to the kind (20). For M=2, we have the general solution (25) to the corresponding Equation (21). Thus, we start from M=3. The equation we have to solve is

(26) $$\frac{dR}{dx}=\alpha_{3}R^{3}+\alpha_{2}R^{2}+\alpha_{1}R+\alpha_{0}.$$

The solution is of the kind (18), and from (19), we have the balanced equation m=1+2L. This fixes the form of the simple equation of Bernoulli for this case: $\frac{dy}{dx}=py+qy^{1+2L}$. We start from the simplest case L=2. The equation of Bernoulli becomes $\frac{dy}{dx}=py+qy^{5}$, and the solution to (26) has the form $R=\beta_{2}y^{2}+\beta_{1}y+\beta_{0}$. The substitution of the last relationships in (26) leads to the following system of nonlinear algebraic relationships

(27) $$\begin{array}{ccc} 2q-\alpha_{3}\beta_{2}^{2} & = & 0\\ \beta_{1}\left(q-3\alpha_{3}\beta_{2}^{2}\right) & = & 0\\ -\alpha_{3}\left[\beta_{0}\beta_{2}^{2}+2\beta_{1}^{2}\beta_{2}+\beta_{2}\left(2\beta_{0}\beta_{2}+\beta_{1}^{2}\right)\right]-\alpha_{2}\beta_{2}^{2} & = & 0\\ \beta_{1}\left[-2\alpha_{2}\beta_{2}-\alpha_{3}\left(6\beta_{0}\beta_{2}+\beta_{1}^{2}\right)\right] & = & 0\\ -\alpha_{3}\left[\beta_{0}\left(2\beta_{0}\beta_{2}+\beta_{1}^{2}\right)+2\beta_{0}\beta_{1}^{2}+\beta_{0}^{2}\beta_{2}\right]-\alpha_{2}\left(2\beta_{0}\beta_{2}+\beta_{1}^{2}\right)-\alpha_{1}\beta_{2} & = & 0\\ \beta_{1}\left(p-3\alpha_{3}\beta_{0}^{2}-2\alpha_{2}\beta_{0}-\alpha_{1}\right) & = & 0\\ \alpha_{3}\beta_{0}^{3}+\alpha_{0}+\alpha_{1}\beta_{0}+\alpha_{2}\beta_{0}^{2} & = & 0 \end{array}$$

The solution to (27) is

(28) $$\begin{array}{c} q=\frac{\alpha_{3}\beta_{2}^{2}}{2};\;\;\;\beta_{1}=0;\;\;\;\beta_{0}=-\frac{\alpha_{2}}{3\alpha_{3}};\;\;\;\alpha_{1}=\frac{\alpha_{2}^{2}}{3\alpha_{3}};\;\;\;\alpha_{0}=\frac{\alpha_{2}^{3}}{27\alpha_{3}^{2}}. \end{array}$$

Thus, the equation

(29) $$\frac{dR}{dx}=\alpha_{3}R^{3}+\alpha_{2}R^{2}+\frac{\alpha_{2}^{2}}{3\alpha_{3}}R+\frac{\alpha_{2}^{3}}{27\alpha_{3}^{2}},$$

has the specific exact analytical solution

(30) $$R\left(x\right)=\sum_{l=0}^{2}\beta_{l}{\left\{\frac{p}{-q+Cp\exp\{-4px\}}\right\}}^{\frac{l}{4}}=-\frac{\alpha_{2}}{3\alpha_{3}}+\beta_{2}{\left\{\frac{p}{-\frac{\alpha_{3}\beta_{2}^{2}}{2}+Cp\exp\left\{-4px\right\}}\right\}}^{\frac{1}{2}}$$

Next, we consider the case M=3, L=3. The equation of Bernoulli becomes $\frac{dy}{dx}=py+qy^{7}$, and the solution to (26) has the form $R=\beta_{3}y^{3}+\beta_{2}y^{2}+\beta_{1}y+\beta_{0}$. The substitution of the last relationships in (26) leads to the following system of nonlinear algebraic relationships

(31) $$\begin{array}{ccc} 3q-\alpha_{3}\beta_{3}^{2} & = & 0\\ \beta_{2}\left(2q-3\alpha_{3}\beta_{3}^{2}\right) & = & 0\\ \beta_{1}q-\alpha_{3}\beta_{3}\left(\beta_{1}\beta_{3}+2\beta_{2}^{2}+2\beta_{1}\beta_{3}+\beta_{2}^{2}\right) & = & 0\\ -\alpha_{2}\beta_{3}^{2}-\alpha_{3}\beta_{3}\left(2\beta_{1}\beta_{2}+3\beta_{0}\beta_{3}+2\beta_{1}\beta_{2}\right)-\alpha_{3}\beta_{2}\left(2\beta_{1}\beta_{3}+\beta_{2}^{2}\right) & = & 0\\ -\alpha_{3}\left[2\beta_{0}\beta_{2}\beta_{3}+\beta_{1}\left(2\beta_{1}\beta_{3}+\beta_{2}^{2}\right)+\beta_{2}\left(2\beta_{0}\beta_{3}+2\beta_{1}\beta_{2}\right)+\beta_{3}\left(2\beta_{0}\beta_{2}+\beta_{1}^{2}\right)\right]-2\alpha_{2}\beta_{2}\beta_{3} & = & 0\\ -\alpha_{3}\left[\beta_{0}\left(2\beta_{1}\beta_{3}+\beta_{2}^{2}\right)+\beta_{1}\left(2\beta_{0}\beta_{3}+2\beta_{1}\beta_{2}\right)+\beta_{2}\left(2\beta_{0}\beta_{2}+\beta_{1}^{2}\right)+2\beta_{0}\beta_{1}\beta_{2}\right]-\\ \alpha_{2}\left(2\beta_{1}\beta_{3}+\beta_{2}^{2}\right) & = & 0\\ 3\beta_{3}p-\alpha_{3}\left[\beta_{1}\left(2\beta_{0}\beta_{2}+\beta_{1}^{2}\right)+\beta_{0}\left(2\beta_{0}\beta_{3}+2\beta_{1}\beta_{2}\right)+\beta_{3}\beta_{0}^{2}+2\beta_{0}\beta_{1}\beta_{2}\right]-\\ \alpha_{2}\left(2\beta_{0}\beta_{3}+2\beta_{1}\beta_{2}\right)-\alpha_{1}\beta_{3} & = & 0\\ -\alpha_{3}\left[\beta_{0}\left(2\beta_{0}\beta_{2}+\beta_{1}^{2}\right)+2\beta_{0}\beta_{1}^{2}+\beta_{2}\beta_{0}^{2}\right]-\alpha_{2}\left(2\beta_{0}\beta_{2}+\beta_{1}^{2}\right)-\alpha_{1}\beta_{2} & = & 0\\ \beta_{1}\left(p-3\alpha_{3}\beta_{0}^{2}-2\alpha_{2}\beta_{0}-\alpha_{1}\right) & = & 0\\ -\alpha_{0}-\alpha_{3}\beta_{0}^{3}-\alpha_{2}\beta_{0}^{2}-\alpha_{1}\beta_{0} & = & 0 \end{array}$$

The solution to (31) is

(32) $$\begin{array}{c} q=-\frac{\alpha_{2}}{3\alpha_{3}};\;\;\;p=\frac{-\alpha_{2}^{2}+3\alpha_{1}\alpha_{3}}{9\alpha_{3}};\;\;\;\beta_{0}=-\frac{\alpha_{2}}{3\alpha_{3}};\;\;\;\beta_{1}=\beta_{2}=0;\;\;\;\alpha_{0}=\frac{\alpha_{2}\left(-2\alpha_{2}^{2}+9\alpha_{1}\alpha_{3}\right)}{27\alpha_{3}^{3}} \end{array}$$

Thus, the equation

(33) $$\frac{dR}{dx}=\alpha_{3}R^{3}+\alpha_{2}R^{2}+\alpha_{1}R+\frac{\alpha_{2}\left(-2\alpha_{2}^{2}+9\alpha_{1}\alpha_{3}\right)}{27\alpha_{3}^{3}},$$

has the specific exact analytical solution

(34) $$\begin{array}{c} R\left(x\right)=\sum_{l=0}^{3}\beta_{l}{\left\{\frac{p}{-q+Cp\exp\{-6px\}}\right\}}^{\frac{l}{6}}=-\frac{\alpha_{2}}{3\alpha_{3}}+\beta_{3}{\left\{\frac{-\alpha_{2}^{2}+3\alpha_{1}\alpha_{3}}{3\alpha_{2}+C\left(-\alpha_{2}^{2}+3\alpha_{1}\alpha_{3}\right)\exp\left\{-6\frac{-\alpha_{2}^{2}+3\alpha_{1}\alpha_{3}}{9\alpha_{3}}x\right\}}\right\}}^{\frac{1}{2}} \end{array}$$

Next, we consider the case M=4. We have to solve the equation

(35) $$\frac{dR}{dx}=\alpha_{4}R^{4}+\alpha_{3}R^{3}+\alpha_{2}R^{2}+\alpha_{1}R+\alpha_{0}.$$

The solution is of the kind (18), and from (19), we have the balanced equation m=1+3L. This fixes the form of the simple equation of Bernoulli for this case: $\frac{dy}{dx}=py+qy^{1+3L}$. We start from the simplest case L=2. The equation of Bernoulli becomes $\frac{dy}{dx}=py+qy^{7}$, and the solution to (35) has the form $R=\beta_{2}y^{2}+\beta_{1}y+\beta_{0}$. The substitution of the last relationships in (35) leads to the following system of nonlinear algebraic relationships

(36) $$\begin{array}{ccc} \beta_{2}\left(2q-\alpha_{4}\beta_{2}^{3}\right) & = & 0\\ \beta_{1}\left(q-4\alpha_{4}\beta_{2}^{3}\right) & = & 0\\ -\beta_{2}^{2}\left[\alpha_{3}\beta_{2}-\alpha_{4}\left(2\beta_{0}\beta_{2}+\beta_{1}^{2}\right)+4\beta_{1}^{2}\right] & = & 0\\ \beta_{1}\left[-3\alpha_{3}\beta_{2}^{2}-\alpha_{4}\left(4\beta_{0}\beta_{2}^{2}+4\beta_{2}\left(2\beta_{0}\beta_{2}+\beta_{1}^{2}\right)\right)\right)] & = & 0\\ -\alpha_{4}\left[2\beta_{0}^{2}\beta_{2}^{2}+8\beta_{0}\beta_{1}^{2}\beta_{2}+{\left(2\beta_{0}\beta_{2}+\beta_{1}^{2}\right)}^{2}\right]-\alpha_{3}\left[\beta_{0}\beta_{2}^{2}+2\beta_{1}^{2}\beta_{2}+\beta_{2}\left(2\beta_{0}\beta_{2}+\beta_{1}^{2}\right)\right]-\alpha_{2}\beta_{2}^{2} & = & 0\\ \beta_{1}\left\{-\alpha_{4}\left[4\beta_{0}^{2}\beta_{2}+4\beta_{0}\left(2\beta_{0}\beta_{2}+\beta_{1}^{2}\right)\right]-\alpha_{3}\left[4\beta_{0}\beta_{2}+2\beta_{0}\beta_{2}+\beta_{1}^{2}\right]-2\alpha_{2}\beta_{2}\right\} & = & 0\\ -\alpha_{4}\beta_{0}^{2}\left(4\beta_{0}\beta_{2}+6\beta_{1}^{2}\right)-\alpha_{3}\beta_{0}\left(2\beta_{0}\beta_{2}+3\beta_{1}^{2}+\beta_{2}\right)-\alpha_{2}\left(2\beta_{0}\beta_{2}+\beta_{1}^{2}\right)-\alpha_{1}\beta_{2} & = & 0\\ \beta_{1}\left(p-4\alpha_{4}\beta_{0}^{3}-3\alpha_{3}\beta_{0}^{2}-2\alpha_{2}\beta_{0}-\alpha_{1}\right) & = & 0\\ -\alpha_{3}\beta_{0}^{3}-\alpha_{0}-\alpha_{2}\beta_{0}^{2}-\alpha_{4}\beta_{0}^{4}-\alpha_{1}\beta_{0} & = & 0 \end{array}$$

The solution to (36) is

(37) $$\begin{array}{c} q=\frac{\alpha_{4}\beta_{2}^{3}}{4};\;\;\;\beta_{0}=-\frac{\alpha_{3}}{4\alpha_{4}};\;\;\;\beta_{1}=0;\;\;\;\alpha_{0}=\frac{\alpha_{3}^{4}}{256\alpha_{4}^{3}};\;\;\;\alpha_{1}=\frac{\alpha_{3}^{2}}{16\alpha_{4}^{2}};\;\;\;\alpha_{2}=\frac{\alpha_{3}^{2}}{8\alpha_{4}} \end{array}$$

Thus, the equation

(38) $$\frac{dR}{dx}=\alpha_{4}R^{4}+\alpha_{3}R^{3}+\frac{\alpha_{3}^{2}}{8\alpha_{4}}R^{2}+\frac{\alpha_{3}^{2}}{16\alpha_{4}^{2}}R+\frac{\alpha_{3}^{4}}{256\alpha_{4}^{3}},$$

has the solution

(39) $$\begin{array}{c} R=\beta_{2}y^{2}+\beta_{1}y+\beta_{0}=-\frac{\alpha_{3}}{4\alpha_{4}}+\beta_{2}{\left\{\frac{p}{-\frac{\alpha_{4}\beta_{2}^{3}}{4}+Cp\exp\left\{-6px\right\}}\right\}}^{\frac{1}{3}} \end{array}$$

Next, we consider the case M=5. We have to solve the equation

(40) $$\frac{dR}{dx}=\alpha_{5}R^{5}+\alpha_{4}R^{4}+\alpha_{3}R^{3}+\alpha_{2}R^{2}+\alpha_{1}R+\alpha_{0}.$$

The solution is of the kind (18), and from (19), we have the balanced equation m=1+4L. This fixes the form of the simple equation of Bernoulli for this case: $\frac{dy}{dx}=py+qy^{1+4L}$. We start from the simplest case L=2. The equation of Bernoulli becomes $\frac{dy}{dx}=py+qy^{9}$, and the solution of (35) has the form $R=\beta_{2}y^{2}+\beta_{1}y+\beta_{0}$. The substitution of the last relationships in (39) leads to the following system of nonlinear algebraic relationships

(41) $$\begin{array}{ccc} \beta_{2}\left(2q-\alpha_{5}\beta_{2}^{4}\right) & = & 0\\ \beta_{1}\left(q-5\alpha_{5}\beta_{2}^{4}\right) & = & 0\\ -\alpha_{5}\beta_{2}\left[5\beta_{0}\beta_{2}^{3}+10\beta_{1}^{2}\beta_{2}^{2}+4\beta_{0}\beta_{1}\beta_{2}^{2}+4\beta_{1}\left(2\beta_{0}\beta_{2}+\beta_{1}^{2}\right)\right] & = & 0\\ \beta_{1}\left[-4\alpha_{4}\beta_{2}^{3}-\alpha_{5}\left(8\beta_{0}\beta_{2}^{3}+4\beta_{1}^{2}\beta_{2}^{2}+4\beta_{1}\beta_{2}^{2}\right)+\beta_{2}\left(12\beta_{0}\beta_{2}^{2}+4\beta_{1}^{2}\beta_{2}\right)\right] & = & 0\\ -\alpha_{5}[2\beta_{0}\beta_{2}^{2}\left(2\beta_{0}\beta_{2}+\beta_{1}^{2}+2\beta_{1}^{2}\right)+\beta_{1}^{2}\beta_{2}\left(12\beta_{0}\beta_{2}+4\beta_{1}^{2}\right)+\\ \beta_{2}\left(2\beta_{0}^{2}\beta_{2}^{2}+16\beta_{0}\beta_{1}^{2}\beta_{2}+2{\left(2\beta_{0}\beta_{2}+\beta_{1}^{2}\right)}^{2}\right]-\alpha_{4}\beta_{2}^{2}\left(4\beta_{0}\beta_{2}+6\beta_{1}^{2}\right)-\alpha_{3}\beta_{2}^{3} & = & 0\\ -\alpha_{5}[4\beta_{0}\beta_{1}\beta_{2}\left(\beta_{0}\beta_{2}+{\left(2\beta_{0}\beta_{2}+\beta_{1}^{2}\right)}^{2}\right)+\beta_{1}\left(2\beta_{0}^{2}\beta_{2}^{2}+8\beta_{0}\beta_{1}^{2}\beta_{2}+{\left(2\beta_{0}\beta_{2}+\beta_{1}^{2}\right)}^{2}\right)+\\ 4\beta_{0}\beta_{1}\beta_{2}\left(\beta_{0}\beta_{2}+\left(2\beta_{0}\beta_{2}+\beta_{1}^{2}\right)\right)]-4\alpha_{4}\beta_{1}\beta_{2}\left(3\beta_{0}\beta_{2}+\beta_{1}^{2}\right)-3\alpha_{3}\beta_{1}\beta_{2}^{2} & = & 0\\ -\alpha_{5}[\beta_{0}\left(2\beta_{0}^{2}\beta_{2}^{2}+8\beta_{0}\beta_{1}^{2}\beta_{2}+{\left(2\beta_{0}\beta_{2}+\beta_{1}^{2}\right)}^{2}\right)+4\beta_{0}\beta_{1}^{2}\left(3\beta_{0}\beta_{2}+\beta_{1}^{2}\right)+\\ 2\beta_{0}^{2}\beta_{2}\left(2\beta_{0}\beta_{2}+3\beta_{1}^{2}\right)]-3\alpha_{4}\beta_{2}\left(\beta_{0}\beta_{2}+\beta_{1}^{2}\right)-3\alpha_{3}\beta_{2}\left(\beta_{0}\beta_{2}+\beta_{1}^{2}\right)-\alpha_{2}\beta_{2}^{2} & = & 0\\ -\alpha_{5}\left[4\beta_{0}^{2}\beta_{1}\left(3\beta_{0}\beta_{2}+\beta_{1}^{2}\right)+\beta_{1}\beta_{0}^{2}\left(4\beta_{0}\beta_{2}+6\beta_{1}^{2}\right)+4\beta_{2}\beta_{0}^{3}\beta_{1}\right]-\\ 4\alpha_{4}\beta_{0}\beta_{1}\left(2\beta_{0}\beta_{2}+\beta_{1}^{2}\right)-\alpha_{3}\beta_{1}\left(6\beta_{0}\beta_{2}+\beta_{1}^{2}\right)-2\alpha_{2}\beta_{1}\beta_{2} & = & 0\\ -\alpha_{5}\beta_{0}^{3}\left(5\beta_{0}\beta_{2}+10\beta_{1}^{2}\right)-\alpha_{4}\beta_{0}^{2}\left(4\beta_{0}\beta_{2}+6\beta_{1}^{2}\right)-2\alpha_{3}\beta_{0}\left(\beta_{0}\beta_{2}+\beta_{1}^{2}\right)-\\ \alpha_{2}\left(\beta_{0}\beta_{2}+\beta_{1}^{2}\right)-\alpha_{1}\beta_{2} & = & 0\\ \beta_{1}p-5\alpha_{5}\beta_{0}^{4}\beta_{1}-4\alpha_{4}\beta_{0}^{3}\beta_{1}-3\alpha_{3}\beta_{0}^{2}\beta_{1}-2\alpha_{2}\beta_{0}\beta_{1}-\alpha_{1}\beta_{1} & = & 0\\ -\alpha_{0}-\alpha_{2}\beta_{0}^{2}-\alpha_{4}\beta_{0}^{4}-\alpha_{3}\beta_{0}^{3}-\alpha_{5}\beta_{0}^{5}-\alpha_{1}\beta_{0} & = & 0 \end{array}$$

The solution to (41) is

(42) $$\begin{array}{c} q=\frac{\alpha_{5}\beta_{2}^{4}}{2};\;\;\;\beta_{0}=-\frac{\alpha_{4}}{5\alpha_{5}};\;\;\;\beta_{1}=0;\;\;\;\alpha_{0}=\frac{\alpha_{4}^{5}}{3125\alpha_{5}^{4}};\;\;\;\alpha_{1}=\frac{\alpha_{4}^{4}}{125\alpha_{5}^{3}};\;\;\;\alpha_{2}=\frac{2\alpha_{4}^{3}}{25\alpha_{5}^{2}};\;\;\;\alpha_{3}=\frac{2\alpha_{4}^{2}}{5\alpha_{5}} \end{array}$$

Thus, the equation

(43) $$\frac{dR}{dx}=\alpha_{5}R^{5}+\alpha_{4}R^{4}+\frac{2\alpha_{4}^{2}}{5\alpha_{5}}R^{3}+\frac{2\alpha_{4}^{3}}{25\alpha_{5}^{2}}R^{2}+\frac{\alpha_{4}^{4}}{125\alpha_{5}^{3}}R+\frac{\alpha_{4}^{5}}{3125\alpha_{5}^{4}},$$

has the specific solution

(44) $$\begin{array}{c} R=\beta_{2}{\left\{\frac{p}{-\frac{\alpha_{5}\beta_{2}^{4}}{2}+Cp\exp\left\{-8px\right\}}\right\}}^{\frac{1}{4}}-\frac{\alpha_{4}}{5\alpha_{5}} \end{array}$$

Obtaining the exact solutions to the chain of equations can be continued. Below we focus on the epidemic waves connected to the SIR model. The additional exact solutions to the chain of equations will be discussed elsewhere.

## 4. Discussion of the Obtained Exact Analytical Solutions to the Studied Chain of Equations from the Point of View of Modeling of Epidemic Waves

Above, we have obtained several exact solutions to equations that can be connected to the SIR model of epidemic waves. The solutions are of two classes: (i) solutions that can be used for the purposes of the SIR model and (ii) solutions that are not appropriate for the use of the purposes of the SIR model. We begin the discussion by considering the solutions that can be used for the purposes of the SIR model. There are two groups of these solutions. The first group contains solutions without relationships among parameters $\alpha_{i}$ in the form of equalities. The second group contains solutions with relationships among parameters $\alpha_{i}$, which are in the form of equalities.

Above, we have obtained relationships for the quantity R(x), where *x* was some coordinate, which, in particular, can be some spatial coordinate, time, or a combination of time and spatial coordinates. Below, we consider the specific case when the coordinate *x* is time *t*. Thus, we obtain solutions to the number of recovered people R(t) for the case of the SIR model. This allows us to calculate the time evolution of infected people *I* on the basis of (8): $I=\frac{1}{\rho }\frac{dR}{dt}$. Then, we can calculate the relative growth rate

(45) $$\sigma \left(t\right)=\frac{1}{I}\frac{dI}{dt}.$$

It can be written as

(46) $$\sigma \left(t\right)=\rho \left(R_{n}-1\right).$$

In (46)

(47) $$R_{n}\left(t\right)=1+\frac{\sigma (t)}{\rho },$$

is the time-varying effective reproduction number. We use $R_{n}$ for the effective reproduction number in order to distinguish it from *R* by which we denote the recovered people. (46) shows that there is a specific value $R_{n}=1$. If $R_{n}<1$, then σ(t)<0, and the relative growth rate is negative. This means that dI/dt is negative; in other words, the number of infected individuals decreases, and the epidemic shrinks. If $R_{n}>1$, then σ(t)>0, and the relative growth rate is positive. This means that dI/dt is positive; in other words, the number of infected individuals increases, and the epidemic extends. Note that we use (47) instead of the exact relationship $R_{n}=\frac{\tau S}{\rho N}$, as we work with an approximate solution of (10).

We stress here the following. Our basic assumption for reducing the SIR model to a chain of equations was ${\left(\frac{\tau R}{\rho N}\right)}^{2}<<1$. In order to keep the assumption in order, we have to consider epidemic waves for which R<<N. This means that the epidemic wave has to affect a small amount of the entire population. If this is not the case, we have to solve the SIR model numerically. For the rest of this section, we assume that we are within the scope of the assumption ${\left(\frac{\tau R}{\rho N}\right)}^{2}<<1$, which allows us to obtain analytical results.

We have analytical relationships for several epidemic waves. Thus, we can calculate their characteristics by means of the relationships mentioned above. We denote $R_{n}\left(0\right)=\frac{\tau S(0)}{\rho N}$. For the calculation of *S*, we will use the approximate relationship that occurs from (9)

(48) $$S\left(t\right)=S\left(0\right)\left[1-\frac{\tau R}{\rho N}\right]$$

We start from the specific solution (22). Taking into account (13) and (46), we obtain

(49) $$\begin{array}{c} R\left(t\right)=\frac{S\left(0\right)[R_{n}\left(0\right)-1]}{2R_{n}{\left(0\right)}^{2}}+\frac{\delta S\left(0\right){\left\{{\left[R_{n}\left(0\right)-1\right]}^{2}+4R_{n}{\left(0\right)}^{2}\frac{N-S(0)}{S(0)}\right\}}^{1/2}}{2R_{n}{\left(0\right)}^{2}}\tanh\{\frac{1}{2}\delta \rho \times \\ {}[{\left(R_{n}\left(0\right)-1\right)}^{2}+4R_{n}{\left(0\right)}^{2}\frac{N-S(0)}{S(0)}{]}^{1/2}\{t+\frac{2\mathrm{artanh}\left[-\frac{R_{n}\left(0\right)-1}{\delta {\left[{\left(R_{n}\left(0\right)-1\right)}^{2}+4R_{n}{\left(0\right)}^{2}\frac{N-S(0)}{S(0)}\right]}^{1/2}}\right]}{\delta \rho {\left[{\left(R_{n}\left(0\right)-1\right)}^{2}+4R_{n}{\left(0\right)}^{2}\frac{N-S(0)}{S(0)}\right]}^{1/2}}\}\}, \end{array}$$

where δ=±1. Equation (47) allows us to calculate the time evolution of infected individuals for this specific solution. From (8), we obtain

(50) $$\begin{array}{c} I\left(t\right)=\frac{1}{\rho }\frac{dR}{dt}=\frac{S(0)}{4R_{n}{\left(0\right)}^{2}}\left\{{\left[R_{n}\left(0\right)-1\right]}^{2}+4R_{n}^{2}\left(0\right)\frac{N-S(0)}{S(0)}\right\}{\mathrm{sech}}^{2}\{\frac{1}{2}\delta \rho \times \\ {\left[{\left(R_{n}\left(0\right)-1\right)}^{2}+4R_{n}{\left(0\right)}^{2}\frac{N-S(0)}{S(0)}\right]}^{1/2}\left\{t+\frac{2\mathrm{artanh}\left[-\frac{R_{n}\left(0\right)-1}{\delta {\left[{\left(R_{n}\left(0\right)-1\right)}^{2}+4R_{n}{\left(0\right)}^{2}\frac{N-S(0)}{S(0)}\right]}^{1/2}}\right]}{\delta \rho {\left[{\left(R_{n}\left(0\right)-1\right)}^{2}+4R_{n}{\left(0\right)}^{2}\frac{N-S(0)}{S(0)}\right]}^{1/2}}\right\}\} \end{array}$$

From (49) and (48), we obtain

(51) $$\begin{array}{c} S\left(t\right)=S\left(0\right)\{1-\frac{\tau }{\rho N}\frac{S\left(0\right)[R_{n}\left(0\right)-1]}{2R_{n}{\left(0\right)}^{2}}+\frac{\delta S\left(0\right){\left\{{\left[R_{n}\left(0\right)-1\right]}^{2}+4R_{n}{\left(0\right)}^{2}\frac{N-S(0)}{S(0)}\right\}}^{1/2}}{2R_{n}{\left(0\right)}^{2}}\tanh\{\frac{1}{2}\delta \rho \times \\ {\left[{\left(R_{n}\left(0\right)-1\right)}^{2}+4R_{n}{\left(0\right)}^{2}\frac{N-S(0)}{S(0)}\right]}^{1/2}\left\{t+\frac{2\mathrm{artanh}\left[-\frac{R_{n}\left(0\right)-1}{\delta {\left[{\left(R_{n}\left(0\right)-1\right)}^{2}+4R_{n}{\left(0\right)}^{2}\frac{N-S(0)}{S(0)}\right]}^{1/2}}\right]}{\delta \rho {\left[{\left(R_{n}\left(0\right)-1\right)}^{2}+4R_{n}{\left(0\right)}^{2}\frac{N-S(0)}{S(0)}\right]}^{1/2}}\right\}\}\}. \end{array}$$

This allows us to calculate σ(t) from (46)

(52) $$\begin{array}{c} \sigma \left(t\right)=\frac{1}{I}\frac{dI}{dt}=-\delta \rho \tanh\{\frac{1}{2}\delta \rho \times \\ {\left[{\left(R_{n}\left(0\right)-1\right)}^{2}+4R_{n}{\left(0\right)}^{2}\frac{N-S(0)}{S(0)}\right]}^{1/2}\left\{t+\frac{2\mathrm{artanh}\left[-\frac{R_{n}\left(0\right)-1}{\delta {\left[{\left(R_{n}\left(0\right)-1\right)}^{2}+4R_{n}{\left(0\right)}^{2}\frac{N-S(0)}{S(0)}\right]}^{1/2}}\right]}{\delta \rho {\left[{\left(R_{n}\left(0\right)-1\right)}^{2}+4R_{n}{\left(0\right)}^{2}\frac{N-S(0)}{S(0)}\right]}^{1/2}}\right\}\}\}. \end{array}$$

Then, from (47)

(53) $$\begin{array}{c} R_{n}\left(t\right)=1-\delta \tanh\{\frac{1}{2}\delta \rho \times \\ {\left[{\left(R_{n}\left(0\right)-1\right)}^{2}+4R_{n}{\left(0\right)}^{2}\frac{N-S(0)}{S(0)}\right]}^{1/2}\left\{t+\frac{2\mathrm{artanh}\left[-\frac{R_{n}\left(0\right)-1}{\delta {\left[{\left(R_{n}\left(0\right)-1\right)}^{2}+4R_{n}{\left(0\right)}^{2}\frac{N-S(0)}{S(0)}\right]}^{1/2}}\right]}{\delta \rho {\left[{\left(R_{n}\left(0\right)-1\right)}^{2}+4R_{n}{\left(0\right)}^{2}\frac{N-S(0)}{S(0)}\right]}^{1/2}}\right\}\}\}. \end{array}$$

We remember that the above results are valid if ${\left(\frac{\tau R}{\rho N}\right)}^{2}<<1$. In other words, we have satisfy

(54) $$\begin{array}{c} \{\frac{\tau }{\rho N}\{\frac{S\left(0\right)[R_{n}\left(0\right)-1]}{2R_{n}{\left(0\right)}^{2}}+\frac{\delta S\left(0\right){\left\{{\left[R_{n}\left(0\right)-1\right]}^{2}+4R_{n}{\left(0\right)}^{2}\frac{N-S(0)}{S(0)}\right\}}^{1/2}}{2R_{n}{\left(0\right)}^{2}}\tanh\{\frac{1}{2}\delta \rho \times \\ {\left[{\left(R_{n}\left(0\right)-1\right)}^{2}+4R_{n}{\left(0\right)}^{2}\frac{N-S(0)}{S(0)}\right]}^{1/2}\left\{t+\frac{2\mathrm{artanh}\left[-\frac{R_{n}\left(0\right)-1}{\delta {\left[{\left(R_{n}\left(0\right)-1\right)}^{2}+4R_{n}{\left(0\right)}^{2}\frac{N-S(0)}{S(0)}\right]}^{1/2}}\right]}{\delta \rho {\left[{\left(R_{n}\left(0\right)-1\right)}^{2}+4R_{n}{\left(0\right)}^{2}\frac{N-S(0)}{S(0)}\right]}^{1/2}}\right\}\}\}{\}}^{2}<<1 \end{array}$$

We can obtain the following estimation for this condition. The maximum value of the tanh function is 1. This, from (14), we obtain

(55) $$\begin{array}{c} \frac{\tau^{2}}{\rho^{2}N^{2}}{\left\{\frac{S\left(0\right)[R_{n}\left(0\right)-1]}{2R_{n}{\left(0\right)}^{2}}+\frac{\delta S\left(0\right){\left\{{\left[R_{n}\left(0\right)-1\right]}^{2}+4R_{n}{\left(0\right)}^{2}\frac{N-S(0)}{S(0)}\right\}}^{1/2}}{2R_{n}{\left(0\right)}^{2}}\right\}}^{2}<<1. \end{array}$$

Next we calculate the quantities for the solution (25). The requirement R(0)=0 leads to the determination of the constant *C* as follows:

(56) $$\begin{array}{c} C=\frac{2}{\delta \rho {\left\{{\left[R_{n}\left(0\right)-1\right]}^{2}+4R_{n}{\left(0\right)}^{2}\frac{N-S(0)}{S(0)}\right\}}^{1/2}}\times \\ \mathrm{artanh}\left\{\frac{\delta {\left\{{\left[R_{n}\left(0\right)-1\right]}^{2}+4R_{n}{\left(0\right)}^{2}\frac{N-S(0)}{S(0)}\right\}}^{1/2}\left\{\frac{-2DR_{n}{\left(0\right)}^{2}}{S(0)}-E\left[R_{n}\left(0\right)-1\right]\right\}}{E\left\{{\left[R_{n}\left(0\right)-1\right]}^{2}+4R_{n}{\left(0\right)}^{2}\frac{N-S(0)}{S(0)}\right\}+\frac{2DR_{n}{\left(0\right)}^{2}}{S(0)}\left[R_{n}\left(0\right)-1\right]}\right\} \end{array}$$

The solution (25) becomes

(57) $$\begin{array}{c} R\left(t\right)=\frac{S\left(0\right)[R_{n}\left(0\right)-1]}{2R_{n}{\left(0\right)}^{2}}+\frac{\delta S\left(0\right){\left\{{\left[R_{n}\left(0\right)-1\right]}^{2}+4R_{n}{\left(0\right)}^{2}\frac{N-S(0)}{S(0)}\right\}}^{1/2}}{2R_{n}{\left(0\right)}^{2}}\tanh\{\frac{1}{2}\delta \rho \times \\ {\left[{\left(R_{n}\left(0\right)-1\right)}^{2}+4R_{n}{\left(0\right)}^{2}\frac{N-S(0)}{S(0)}\right]}^{1/2}\{t+\frac{2}{\delta \rho {\left\{{\left[R_{n}\left(0\right)-1\right]}^{2}+4R_{n}{\left(0\right)}^{2}\frac{N-S(0)}{S(0)}\right\}}^{1/2}}\times \\ \mathrm{artanh}\left\{\frac{\delta {\left\{{\left[R_{n}\left(0\right)-1\right]}^{2}+4R_{n}{\left(0\right)}^{2}\frac{N-S(0)}{S(0)}\right\}}^{1/2}\left\{\frac{-2DR_{n}{\left(0\right)}^{2}}{S(0)}-E\left[R_{n}\left(0\right)-1\right]\right\}}{E\left\{{\left[R_{n}\left(0\right)-1\right]}^{2}+4R_{n}{\left(0\right)}^{2}\frac{N-S(0)}{S(0)}\right\}+\frac{2DR_{n}{\left(0\right)}^{2}}{S(0)}\left[R_{n}\left(0\right)-1\right]}\right\}\}\}+\\ D/\{\cosh^{2}\{\frac{1}{2}\delta \rho \times \\ {\left[{\left(R_{n}\left(0\right)-1\right)}^{2}+4R_{n}{\left(0\right)}^{2}\frac{N-S(0)}{S(0)}\right]}^{1/2}\{t+\frac{2}{\delta \rho {\left\{{\left[R_{n}\left(0\right)-1\right]}^{2}+4R_{n}{\left(0\right)}^{2}\frac{N-S(0)}{S(0)}\right\}}^{1/2}}\times \\ \mathrm{artanh}\left\{\frac{\delta {\left\{{\left[R_{n}\left(0\right)-1\right]}^{2}+4R_{n}{\left(0\right)}^{2}\frac{N-S(0)}{S(0)}\right\}}^{1/2}\left\{\frac{-2DR_{n}{\left(0\right)}^{2}}{S(0)}-E\left[R_{n}\left(0\right)-1\right]\right\}}{E\left\{{\left[R_{n}\left(0\right)-1\right]}^{2}+4R_{n}{\left(0\right)}^{2}\frac{N-S(0)}{S(0)}\right\}+\frac{2DR_{n}{\left(0\right)}^{2}}{S(0)}\left[R_{n}\left(0\right)-1\right]}\right\}\}\}\{E+\\ \frac{2DR_{n}{\left(0\right)}^{2}}{\delta S\left(0\right){\left[{\left(R_{n}\left(0\right)-1\right)}^{2}+4R_{n}{\left(0\right)}^{2}\frac{N-S(0)}{S(0)}\right]}^{1/2}}\tanh\{\frac{1}{2}\delta \rho \times \\ {\left[{\left(R_{n}\left(0\right)-1\right)}^{2}+4R_{n}{\left(0\right)}^{2}\frac{N-S(0)}{S(0)}\right]}^{1/2}\{t+\frac{2}{\delta \rho {\left\{{\left[R_{n}\left(0\right)-1\right]}^{2}+4R_{n}{\left(0\right)}^{2}\frac{N-S(0)}{S(0)}\right\}}^{1/2}}\times \\ \mathrm{artanh}\left\{\frac{\delta {\left\{{\left[R_{n}\left(0\right)-1\right]}^{2}+4R_{n}{\left(0\right)}^{2}\frac{N-S(0)}{S(0)}\right\}}^{1/2}\left\{\frac{-2DR_{n}{\left(0\right)}^{2}}{S(0)}-E\left[R_{n}\left(0\right)-1\right]\right\}}{E\left\{{\left[R_{n}\left(0\right)-1\right]}^{2}+4R_{n}{\left(0\right)}^{2}\frac{N-S(0)}{S(0)}\right\}+\frac{2DR_{n}{\left(0\right)}^{2}}{S(0)}\left[R_{n}\left(0\right)-1\right]}\right\}\}\}\}\}. \end{array}$$

In (57) δ=±1. Equation (57) allows us to calculate the other quantities connected to this solution as follows

$$\begin{array}{c} I=\frac{1}{\rho }\frac{dR}{dt}=\frac{S(0)}{4R_{n}{\left(0\right)}^{2}}\left\{{\left[R_{n}\left(0\right)-1\right]}^{2}+4R_{n}{\left(0\right)}^{2}\frac{N-S(0)}{S(0)}\right\}\times \\ {\mathrm{sech}}^{2}\{\frac{1}{2}\delta \rho {\left[{\left(R_{n}\left(0\right)-1\right)}^{2}+4R_{n}{\left(0\right)}^{2}\frac{N-S(0)}{S(0)}\right]}^{1/2}\{t+\\ \frac{2}{\delta \rho {\left\{{\left[R_{n}\left(0\right)-1\right]}^{2}+4R_{n}{\left(0\right)}^{2}\frac{N-S(0)}{S(0)}\right\}}^{1/2}}\times \\ \mathrm{artanh}\left\{\frac{\delta {\left\{{\left[R_{n}\left(0\right)-1\right]}^{2}+4R_{n}{\left(0\right)}^{2}\frac{N-S(0)}{S(0)}\right\}}^{1/2}\left\{\frac{-2DR_{n}{\left(0\right)}^{2}}{S(0)}-E\left[R_{n}\left(0\right)-1\right]\right\}}{E\left\{{\left[R_{n}\left(0\right)-1\right]}^{2}+4R_{n}{\left(0\right)}^{2}\frac{N-S(0)}{S(0)}\right\}+\frac{2DR_{n}{\left(0\right)}^{2}}{S(0)}\left[R_{n}\left(0\right)-1\right]}\right\}\}\}\}-\\ \delta D{\left[{\left(R_{n}\left(0\right)-1\right)}^{2}+4R_{n}{\left(0\right)}^{2}\frac{N-S(0)}{S(0)}\right]}^{1/2}/\{\cosh^{3}\{\frac{1}{2}\delta \rho {\left[{\left(R_{n}\left(0\right)-1\right)}^{2}+4R_{n}{\left(0\right)}^{2}\frac{N-S(0)}{S(0)}\right]}^{1/2}\{t+\\ \frac{2}{\delta \rho {\left\{{\left[R_{n}\left(0\right)-1\right]}^{2}+4R_{n}{\left(0\right)}^{2}\frac{N-S(0)}{S(0)}\right\}}^{1/2}}\times \\ \mathrm{artanh}\left\{\frac{\delta {\left\{{\left[R_{n}\left(0\right)-1\right]}^{2}+4R_{n}{\left(0\right)}^{2}\frac{N-S(0)}{S(0)}\right\}}^{1/2}\left\{\frac{-2DR_{n}{\left(0\right)}^{2}}{S(0)}-E\left[R_{n}\left(0\right)-1\right]\right\}}{E\left\{{\left[R_{n}\left(0\right)-1\right]}^{2}+4R_{n}{\left(0\right)}^{2}\frac{N-S(0)}{S(0)}\right\}+\frac{2DR_{n}{\left(0\right)}^{2}}{S(0)}\left[R_{n}\left(0\right)-1\right]}\right\}\}\}\{E+\\ \frac{2DR_{n}{\left(0\right)}^{2}}{\delta S\left(0\right){\left[{\left(R_{n}\left(0\right)-1\right)}^{2}+4R_{n}{\left(0\right)}^{2}\frac{N-S(0)}{S(0)}\right]}^{1/2}}\tanh\{\frac{1}{2}\delta \rho {\left[{\left(R_{n}\left(0\right)-1\right)}^{2}+4R_{n}{\left(0\right)}^{2}\frac{N-S(0)}{S(0)}\right]}^{1/2}\{t+\\ \frac{2}{\delta \rho {\left\{{\left[R_{n}\left(0\right)-1\right]}^{2}+4R_{n}{\left(0\right)}^{2}\frac{N-S(0)}{S(0)}\right\}}^{1/2}}\times \\ \mathrm{artanh}\left\{\frac{\delta {\left\{{\left[R_{n}\left(0\right)-1\right]}^{2}+4R_{n}{\left(0\right)}^{2}\frac{N-S(0)}{S(0)}\right\}}^{1/2}\left\{\frac{-2DR_{n}{\left(0\right)}^{2}}{S(0)}-E\left[R_{n}\left(0\right)-1\right]\right\}}{E\left\{{\left[R_{n}\left(0\right)-1\right]}^{2}+4R_{n}{\left(0\right)}^{2}\frac{N-S(0)}{S(0)}\right\}+\frac{2DR_{n}{\left(0\right)}^{2}}{S(0)}\left[R_{n}\left(0\right)-1\right]}\right\}\}\}\}\}-\\ \frac{D^{2}R_{n}{\left(0\right)}^{2}}{S(0)}{\mathrm{sech}}^{2}\{\frac{1}{2}\delta \rho {\left[{\left(R_{n}\left(0\right)-1\right)}^{2}+4R_{n}{\left(0\right)}^{2}\frac{N-S(0)}{S(0)}\right]}^{1/2}\{t+\\ \frac{2}{\delta \rho {\left\{{\left[R_{n}\left(0\right)-1\right]}^{2}+4R_{n}{\left(0\right)}^{2}\frac{N-S(0)}{S(0)}\right\}}^{1/2}}\times \\ \mathrm{artanh}\left\{\frac{\delta {\left\{{\left[R_{n}\left(0\right)-1\right]}^{2}+4R_{n}{\left(0\right)}^{2}\frac{N-S(0)}{S(0)}\right\}}^{1/2}\left\{\frac{-2DR_{n}{\left(0\right)}^{2}}{S(0)}-E\left[R_{n}\left(0\right)-1\right]\right\}}{E\left\{{\left[R_{n}\left(0\right)-1\right]}^{2}+4R_{n}{\left(0\right)}^{2}\frac{N-S(0)}{S(0)}\right\}+\frac{2DR_{n}{\left(0\right)}^{2}}{S(0)}\left[R_{n}\left(0\right)-1\right]}\right\}\}\}\}/\\ \{\cosh^{2}\{\frac{1}{2}\delta \rho {\left[{\left(R_{n}\left(0\right)-1\right)}^{2}+4R_{n}{\left(0\right)}^{2}\frac{N-S(0)}{S(0)}\right]}^{1/2}\{t+\\ \frac{2}{\delta \rho {\left\{{\left[R_{n}\left(0\right)-1\right]}^{2}+4R_{n}{\left(0\right)}^{2}\frac{N-S(0)}{S(0)}\right\}}^{1/2}}\times \\ \mathrm{artanh}\left\{\frac{\delta {\left\{{\left[R_{n}\left(0\right)-1\right]}^{2}+4R_{n}{\left(0\right)}^{2}\frac{N-S(0)}{S(0)}\right\}}^{1/2}\left\{\frac{-2DR_{n}{\left(0\right)}^{2}}{S(0)}-E\left[R_{n}\left(0\right)-1\right]\right\}}{E\left\{{\left[R_{n}\left(0\right)-1\right]}^{2}+4R_{n}{\left(0\right)}^{2}\frac{N-S(0)}{S(0)}\right\}+\frac{2DR_{n}{\left(0\right)}^{2}}{S(0)}\left[R_{n}\left(0\right)-1\right]}\right\}\}\}\{E+\\ \frac{2DR_{n}{\left(0\right)}^{2}}{\delta S\left(0\right){\left[{\left(R_{n}\left(0\right)-1\right)}^{2}+4R_{n}{\left(0\right)}^{2}\frac{N-S(0)}{S(0)}\right]}^{1/2}}\tanh^{2}\{\frac{1}{2}\delta \rho {\left[{\left(R_{n}\left(0\right)-1\right)}^{2}+4R_{n}{\left(0\right)}^{2}\frac{N-S(0)}{S(0)}\right]}^{1/2}\{t+ \end{array}$$

(58) $$\begin{array}{c} \frac{2}{\delta \rho {\left\{{\left[R_{n}\left(0\right)-1\right]}^{2}+4R_{n}{\left(0\right)}^{2}\frac{N-S(0)}{S(0)}\right\}}^{1/2}}\times \\ \mathrm{artanh}\left\{\frac{\delta {\left\{{\left[R_{n}\left(0\right)-1\right]}^{2}+4R_{n}{\left(0\right)}^{2}\frac{N-S(0)}{S(0)}\right\}}^{1/2}\left\{\frac{-2DR_{n}{\left(0\right)}^{2}}{S(0)}-E\left[R_{n}\left(0\right)-1\right]\right\}}{E\left\{{\left[R_{n}\left(0\right)-1\right]}^{2}+4R_{n}{\left(0\right)}^{2}\frac{N-S(0)}{S(0)}\right\}+\frac{2DR_{n}{\left(0\right)}^{2}}{S(0)}\left[R_{n}\left(0\right)-1\right]}\right\}\}\}\}\}. \end{array}$$

Thus,

(59) $$\begin{array}{c} S\left(t\right)=S\left(0\right)\{1-\frac{\tau }{\rho N}\{\frac{S\left(0\right)[R_{n}\left(0\right)-1]}{2R_{n}{\left(0\right)}^{2}}+\frac{\delta S\left(0\right){\left\{{\left[R_{n}\left(0\right)-1\right]}^{2}+4R_{n}{\left(0\right)}^{2}\frac{N-S(0)}{S(0)}\right\}}^{1/2}}{2R_{n}{\left(0\right)}^{2}}\tanh\{\frac{1}{2}\delta \rho \times \\ {\left[{\left(R_{n}\left(0\right)-1\right)}^{2}+4R_{n}{\left(0\right)}^{2}\frac{N-S(0)}{S(0)}\right]}^{1/2}\{t+\frac{2}{\delta \rho {\left\{{\left[R_{n}\left(0\right)-1\right]}^{2}+4R_{n}{\left(0\right)}^{2}\frac{N-S(0)}{S(0)}\right\}}^{1/2}}\times \\ \mathrm{artanh}\left\{\frac{\delta {\left\{{\left[R_{n}\left(0\right)-1\right]}^{2}+4R_{n}{\left(0\right)}^{2}\frac{N-S(0)}{S(0)}\right\}}^{1/2}\left\{\frac{-2DR_{n}{\left(0\right)}^{2}}{S(0)}-E\left[R_{n}\left(0\right)-1\right]\right\}}{E\left\{{\left[R_{n}\left(0\right)-1\right]}^{2}+4R_{n}{\left(0\right)}^{2}\frac{N-S(0)}{S(0)}\right\}+\frac{2DR_{n}{\left(0\right)}^{2}}{S(0)}\left[R_{n}\left(0\right)-1\right]}\right\}\}\}+\\ D/\{\cosh^{2}\{\frac{1}{2}\delta \rho \times \\ {\left[{\left(R_{n}\left(0\right)-1\right)}^{2}+4R_{n}{\left(0\right)}^{2}\frac{N-S(0)}{S(0)}\right]}^{1/2}\{t+\frac{2}{\delta \rho {\left\{{\left[R_{n}\left(0\right)-1\right]}^{2}+4R_{n}{\left(0\right)}^{2}\frac{N-S(0)}{S(0)}\right\}}^{1/2}}\times \\ \mathrm{artanh}\left\{\frac{\delta {\left\{{\left[R_{n}\left(0\right)-1\right]}^{2}+4R_{n}{\left(0\right)}^{2}\frac{N-S(0)}{S(0)}\right\}}^{1/2}\left\{\frac{-2DR_{n}{\left(0\right)}^{2}}{S(0)}-E\left[R_{n}\left(0\right)-1\right]\right\}}{E\left\{{\left[R_{n}\left(0\right)-1\right]}^{2}+4R_{n}{\left(0\right)}^{2}\frac{N-S(0)}{S(0)}\right\}+\frac{2DR_{n}{\left(0\right)}^{2}}{S(0)}\left[R_{n}\left(0\right)-1\right]}\right\}\}\}\{E+\\ \frac{2DR_{n}{\left(0\right)}^{2}}{\delta S\left(0\right){\left[{\left(R_{n}\left(0\right)-1\right)}^{2}+4R_{n}{\left(0\right)}^{2}\frac{N-S(0)}{S(0)}\right]}^{1/2}}\tanh\{\frac{1}{2}\delta \rho \times \\ {\left[{\left(R_{n}\left(0\right)-1\right)}^{2}+4R_{n}{\left(0\right)}^{2}\frac{N-S(0)}{S(0)}\right]}^{1/2}\{t+\frac{2}{\delta \rho {\left\{{\left[R_{n}\left(0\right)-1\right]}^{2}+4R_{n}{\left(0\right)}^{2}\frac{N-S(0)}{S(0)}\right\}}^{1/2}}\times \\ \mathrm{artanh}\left\{\frac{\delta {\left\{{\left[R_{n}\left(0\right)-1\right]}^{2}+4R_{n}{\left(0\right)}^{2}\frac{N-S(0)}{S(0)}\right\}}^{1/2}\left\{\frac{-2DR_{n}{\left(0\right)}^{2}}{S(0)}-E\left[R_{n}\left(0\right)-1\right]\right\}}{E\left\{{\left[R_{n}\left(0\right)-1\right]}^{2}+4R_{n}{\left(0\right)}^{2}\frac{N-S(0)}{S(0)}\right\}+\frac{2DR_{n}{\left(0\right)}^{2}}{S(0)}\left[R_{n}\left(0\right)-1\right]}\right\}\}\}\}\}\}\} \end{array}$$

Moreover,

(60) $$\begin{array}{c} \sigma \left(t\right)=\{\frac{\rho S\left(0\right)T_{1}^{3/2}\tanh\left(T_{2}\right)\left[1-\tanh^{2}\left(T_{2}\right)\right]}{4R_{n}{\left(0\right)}^{2}}+\frac{3D\rho T_{1}\sinh^{2}\left(T_{2}\right)}{2T_{3}\cosh^{4}\left(T_{2}\right)}+\\ \frac{2\delta D^{2}\rho R_{n}{\left(0\right)}^{2}T_{1}^{1/2}\sinh\left(T_{2}\right)\left[1-\tanh^{2}\left(T_{2}\right)\right]}{S\left(0\right)T_{3}^{2}\cosh^{2}\left(T_{2}\right)}-\frac{\rho DT_{1}}{2T_{3}\cosh^{2}\left(T_{2}\right)}+\frac{2\rho D^{3}R_{n}{\left(0\right)}^{4}\left[1-\tanh^{2}\left(T_{2}\right)\right]}{S{\left(0\right)}^{2}T_{3}^{3}\cosh^{2}\left(T_{2}\right)}+\\ \frac{\delta \rho D^{2}R_{n}{\left(0\right)}^{2}T_{1}^{1/2}\tanh\left(T_{2}\right)\left[1-\tanh^{2}\left(T_{2}\right)\right]}{S\left(0\right)T_{3}^{2}\cosh^{2}\left(T_{2}\right)}\}/\{\frac{S\left(0\right)T_{1}\left[1-\tanh^{2}\left(T_{2}\right)\right]}{4R_{n}{\left(0\right)}^{2}}-\frac{\delta DT_{1}^{1/2}\sinh\left(T_{2}\right)}{T_{3}\cosh^{3}\left(T_{2}\right)}-\\ \frac{D^{2}R_{n}{\left(0\right)}^{2}\left[1-\tanh^{2}\left(T_{2}\right)\right]}{S\left(0\right)T_{3}^{2}\cosh^{2}\left(T_{2}\right)}\} \end{array}$$

where

(61) $$\begin{array}{ccc} T_{1} & = & {\left[R_{n}\left(0\right)-1\right]}^{2}+4\frac{R_{n}{\left(0\right)}^{2}\left[N-S\left(0\right)\right]}{S(0)},\\ T_{2} & = & \frac{1}{2}\delta \rho {\left\{{\left[R_{n}\left(0\right)-1\right]}^{2}+4\frac{R_{n}{\left(0\right)}^{2}\left[N-S\left(0\right)\right]}{S(0)}\right\}}^{1/2}\{t+\frac{2}{\delta \rho {\left\{{\left[R_{n}\left(0\right)-1\right]}^{2}+4\frac{R_{n}{\left(0\right)}^{2}\left[N-S\left(0\right)\right]}{S(0)}\right\}}^{1/2}}\times \\ & & \mathrm{artanh}\left\{\frac{\delta {\left\{{\left[R_{n}\left(0\right)-1\right]}^{2}+4R_{n}{\left(0\right)}^{2}\frac{N-S(0)}{S(0)}\right\}}^{1/2}\left\{\frac{-2DR_{n}{\left(0\right)}^{2}}{S(0)}-E\left[R_{n}\left(0\right)-1\right]\right\}}{E\left\{{\left[R_{n}\left(0\right)-1\right]}^{2}+4R_{n}{\left(0\right)}^{2}\frac{N-S(0)}{S(0)}\right\}+\frac{2DR_{n}{\left(0\right)}^{2}}{S(0)}\left[R_{n}\left(0\right)-1\right]}\right\}\},\\ T_{3} & = & E+2\frac{R_{n}{\left(0\right)}^{2}D\tanh\left(T_{2}\right)}{\delta S\left(0\right)T_{1}^{1/2}}. \end{array}$$

Finally,

(62) $$\begin{array}{c} R_{n}\left(t\right)=1+\{\frac{S\left(0\right)T_{1}^{3/2}\tanh\left(T_{2}\right)\left[1-\tanh^{2}\left(T_{2}\right)\right]}{4R_{n}{\left(0\right)}^{2}}+\frac{3DT_{1}\sinh^{2}\left(T_{2}\right)}{2T_{3}\cosh^{4}\left(T_{2}\right)}+\\ \frac{2\delta D^{2}R_{n}{\left(0\right)}^{2}T_{1}^{1/2}\sinh\left(T_{2}\right)\left[1-\tanh^{2}\left(T_{2}\right)\right]}{S\left(0\right)T_{3}^{2}\cosh^{2}\left(T_{2}\right)}-\frac{DT_{1}}{2T_{3}\cosh^{2}\left(T_{2}\right)}+\frac{2D^{3}R_{n}{\left(0\right)}^{4}\left[1-\tanh^{2}\left(T_{2}\right)\right]}{S{\left(0\right)}^{2}T_{3}^{3}\cosh^{2}\left(T_{2}\right)}+\\ \frac{\delta D^{2}R_{n}{\left(0\right)}^{2}T_{1}^{1/2}\tanh\left(T_{2}\right)\left[1-\tanh^{2}\left(T_{2}\right)\right]}{S\left(0\right)T_{3}^{2}\cosh^{2}\left(T_{2}\right)}\}/\{\frac{S\left(0\right)T_{1}\left[1-\tanh^{2}\left(T_{2}\right)\right]}{4R_{n}{\left(0\right)}^{2}}-\frac{\delta DT_{1}^{1/2}\sinh\left(T_{2}\right)}{T_{3}\cosh^{3}\left(T_{2}\right)}-\\ \frac{D^{2}R_{n}{\left(0\right)}^{2}\left[1-\tanh^{2}\left(T_{2}\right)\right]}{S\left(0\right)T_{3}^{2}\cosh^{2}\left(T_{2}\right)}\} \end{array}$$

The above results are valid if ${\left(\frac{\tau R}{\rho N}\right)}^{2}<<1$. This means that

(63) $$\begin{array}{c} \frac{\tau^{2}}{\rho^{2}N^{2}}\{\frac{S\left(0\right)[R_{n}\left(0\right)-1]}{2R_{n}{\left(0\right)}^{2}}+\frac{\delta S\left(0\right){\left\{{\left[R_{n}\left(0\right)-1\right]}^{2}+4R_{n}{\left(0\right)}^{2}\frac{N-S(0)}{S(0)}\right\}}^{1/2}}{2R_{n}{\left(0\right)}^{2}}\tanh\{\frac{1}{2}\delta \rho \times \\ {\left[{\left(R_{n}\left(0\right)-1\right)}^{2}+4R_{n}{\left(0\right)}^{2}\frac{N-S(0)}{S(0)}\right]}^{1/2}\{t+\frac{2}{\delta \rho {\left\{{\left[R_{n}\left(0\right)-1\right]}^{2}+4R_{n}{\left(0\right)}^{2}\frac{N-S(0)}{S(0)}\right\}}^{1/2}}\times \\ \mathrm{artanh}\left\{\frac{\delta {\left\{{\left[R_{n}\left(0\right)-1\right]}^{2}+4R_{n}{\left(0\right)}^{2}\frac{N-S(0)}{S(0)}\right\}}^{1/2}\left\{\frac{-2DR_{n}{\left(0\right)}^{2}}{S(0)}-E\left[R_{n}\left(0\right)-1\right]\right\}}{E\left\{{\left[R_{n}\left(0\right)-1\right]}^{2}+4R_{n}{\left(0\right)}^{2}\frac{N-S(0)}{S(0)}\right\}+\frac{2DR_{n}{\left(0\right)}^{2}}{S(0)}\left[R_{n}\left(0\right)-1\right]}\right\}\}\}+\\ D/\{\cosh^{2}\{\frac{1}{2}\delta \rho \times \\ {\left[{\left(R_{n}\left(0\right)-1\right)}^{2}+4R_{n}{\left(0\right)}^{2}\frac{N-S(0)}{S(0)}\right]}^{1/2}\{t+\frac{2}{\delta \rho {\left\{{\left[R_{n}\left(0\right)-1\right]}^{2}+4R_{n}{\left(0\right)}^{2}\frac{N-S(0)}{S(0)}\right\}}^{1/2}}\times \\ \mathrm{artanh}\left\{\frac{\delta {\left\{{\left[R_{n}\left(0\right)-1\right]}^{2}+4R_{n}{\left(0\right)}^{2}\frac{N-S(0)}{S(0)}\right\}}^{1/2}\left\{\frac{-2DR_{n}{\left(0\right)}^{2}}{S(0)}-E\left[R_{n}\left(0\right)-1\right]\right\}}{E\left\{{\left[R_{n}\left(0\right)-1\right]}^{2}+4R_{n}{\left(0\right)}^{2}\frac{N-S(0)}{S(0)}\right\}+\frac{2DR_{n}{\left(0\right)}^{2}}{S(0)}\left[R_{n}\left(0\right)-1\right]}\right\}\}\}\{E+\\ \frac{2DR_{n}{\left(0\right)}^{2}}{\delta S\left(0\right){\left[{\left(R_{n}\left(0\right)-1\right)}^{2}+4R_{n}{\left(0\right)}^{2}\frac{N-S(0)}{S(0)}\right]}^{1/2}}\tanh\{\frac{1}{2}\delta \rho \times \\ {\left[{\left(R_{n}\left(0\right)-1\right)}^{2}+4R_{n}{\left(0\right)}^{2}\frac{N-S(0)}{S(0)}\right]}^{1/2}\{t+\frac{2}{\delta \rho {\left\{{\left[R_{n}\left(0\right)-1\right]}^{2}+4R_{n}{\left(0\right)}^{2}\frac{N-S(0)}{S(0)}\right\}}^{1/2}}\times \\ \mathrm{artanh}\left\{\frac{\delta {\left\{{\left[R_{n}\left(0\right)-1\right]}^{2}+4R_{n}{\left(0\right)}^{2}\frac{N-S(0)}{S(0)}\right\}}^{1/2}\left\{\frac{-2DR_{n}{\left(0\right)}^{2}}{S(0)}-E\left[R_{n}\left(0\right)-1\right]\right\}}{E\left\{{\left[R_{n}\left(0\right)-1\right]}^{2}+4R_{n}{\left(0\right)}^{2}\frac{N-S(0)}{S(0)}\right\}+\frac{2DR_{n}{\left(0\right)}^{2}}{S(0)}\left[R_{n}\left(0\right)-1\right]}\right\}\}\}\}\}.{\}}^{2}<<1 \end{array}$$

Next, we discuss the solution (34). For this solution, we have a relationship (28) for $\alpha_{0}$. From the point of view of the SIR model, $\alpha_{0}>0$, $\alpha_{2}<0$ and $\alpha_{3}>0$. In order to ensure that $\alpha_{0}>0$, we see from (28) that it is necessary that $9\alpha_{1}\alpha_{3}-2\alpha_{2}^{2}<0$ This leads to the requirement

(64) $$R_{n}\left(0\right)<9/7.$$

We note that 9/7 is close to 1.3, which was a characteristic empirical value for the strong spreading of the virus in the case of the COVID-19 pandemic in recent years. Taking into account condition (64), we proceed with solution (34). The relationship (28) among the parameters $\alpha_{i}$ fixes one of the parameters of the SIR model. Let us choose to fix S(0). Then, we obtain

(65) $$S\left(0\right)=\frac{N\left\{7\rho^{2}N^{2}+27\tau^{3}+\delta {\left[49\rho^{4}N^{4}+378\rho^{2}N^{2}\tau^{3}+729\tau^{6}-972\tau^{2}\rho^{3}N^{2}\right]}^{1/2}\right\}}{54\tau^{3}},$$

where δ=±1. From (64), it follows that

(66) $$\frac{\left\{7\rho^{2}N^{2}+27\tau^{3}+\delta {\left[49\rho^{4}N^{4}+378\rho^{2}N^{2}\tau^{3}+729\tau^{6}-972\tau^{2}\rho^{3}N^{2}\right]}^{1/2}\right\}}{54\rho \tau^{2}}<9/7.$$

Below, we will write the solutions to δ=−1 as an example.

Another condition is R(0)=0, which fixes the value of the constant *C* to

(67) $$\begin{array}{c} C=\{9\tau [7\tau \beta_{3}^{2}\rho^{2}N^{2}+27\tau^{4}\beta_{3}^{2}-\tau \beta_{3}^{2}{\left(49\rho^{4}N^{4}+378\rho^{2}N^{2}\tau^{3}+729\tau^{6}-972\tau^{2}\rho^{3}N^{2}\right)}^{1/2}-\\ 81\tau^{3}\rho \beta_{3}^{2}+9\rho^{3}N^{3}]\}/\{\rho^{2}N^{2}[7\rho^{2}N^{2}+27\tau^{3}-{\left(49\rho^{4}N^{4}+378\rho^{2}N^{2}\tau^{3}+729\tau^{6}-972\tau^{2}\rho^{3}N^{2}\right)}^{1/2}-\\ 81\rho \tau^{2}]\}. \end{array}$$

Let us define

$$\begin{array}{ccc} T_{1} & = & 49\rho^{4}N^{4}+378\rho^{2}N^{2}\tau^{3}+729\tau^{6}-972\tau^{2}\rho^{3}N^{2};\;\;\;T_{2}=7\rho^{2}N^{2}+27\tau^{3}-T_{1}^{1/2}\\ T_{3} & = & -\frac{T_{2}^{2}}{2916\tau^{2}\rho^{2}N^{2}}+\frac{T_{2}\left[\frac{T_{2}}{54\tau^{2}}-\rho \right]}{18\rho^{2}N^{2}} \end{array}$$

Then, the solution (34) becomes

(68) $$\begin{array}{c} R\left(t\right)=\frac{\rho N}{3\tau }+\beta_{3}\{T3/[[-\frac{T_{2}}{18\tau \rho N}+9\tau T_{3}(7\tau \beta_{3}^{2}\rho^{2}N^{2}+27\tau^{4}\beta_{3}^{2}-\tau \beta_{3}^{2}T_{1}^{1/2}-81\tau^{3}\rho \beta_{3}^{2}+\\ 9\rho^{3}N^{3})\exp\left(-36\frac{\rho^{2}N^{2}T_{3}}{T_{2}}t\right)]/\left(\rho^{2}N^{2}\left(7\rho^{2}N^{2}+27\tau^{3}-T_{1}^{1/2}-81\rho \tau^{2}\right)\right)]{\}}^{1/2} \end{array}$$

Then,

(69) $$\begin{array}{c} I=162\beta_{3}T_{3}^{3}T_{5}\tau \exp\left(-36\frac{\rho^{2}N^{2}T_{3}}{T_{2}}t\right)/\{\rho T_{2}T_{4}{\left[-\frac{T_{2}}{18\tau \rho N}+9\frac{\tau T_{3}T_{5}\exp\left(-36\frac{\rho^{2}N^{2}T_{3}}{T_{2}}t\right)}{\rho^{2}N^{2}T_{4}}\right]}^{2}\times \\ {\left[\frac{T_{3}}{-\frac{T_{2}}{18\tau \rho N}+9\frac{\tau T_{3}T_{5}\exp\left(-36\frac{\rho^{2}N^{2}T_{3}}{T_{2}}t\right)}{\rho^{2}N^{2}T_{4}}}\right]}^{1/2}\}. \end{array}$$

Here, $T_{1,2,3}$ are as above and

$$\begin{array}{c} T_{4}=T_{2}-81\rho \tau^{2};\;\;\;T_{5}=27\tau^{4}\beta_{3}^{2}+7\tau \beta_{3}^{2}\rho^{2}N^{2}-\tau \beta_{3}^{2}T_{1}^{1/2}-81\tau^{3}\rho \beta_{3}^{2}+9\rho^{3}N^{3}. \end{array}$$

Furthermore,

(70) $$\begin{array}{c} \sigma \left(t\right)=\rho {\left(T_{3}/T_{6}\right)}^{1/2}T_{2}T_{4}T_{6}^{2}[-26244\frac{\beta_{3}\tau^{2}T_{3}^{6}T_{5}^{2}T_{7}^{2}}{\rho T_{2}^{2}T_{4}^{2}T_{6}^{4}{\left(T_{3}/T_{6}\right)}^{3/2}}+104976\frac{\beta_{3}\tau^{2}T_{3}^{5}T_{5}^{2}T_{7}^{2}}{\rho T_{2}^{2}T_{4}^{2}T_{6}^{3}{\left(T_{3}/T_{6}\right)}^{1/2}}-\\ 5832\frac{\beta_{3}\rho \tau N^{2}T_{3}^{4}T_{5}T_{7}}{T_{2}^{2}T_{4}T_{6}^{2}{\left(T_{3}/T_{6}\right)}^{1/2}}]/\left(162\beta_{3}\tau T_{3}^{3}T_{5}T_{7}\right). \end{array}$$

Here $T_{1},T_{2},T_{3},T_{4},T_{5}$ are as above and

$$\begin{array}{c} T_{6}=-\frac{T_{2}}{18\tau \rho N}+9\frac{\tau T_{3}T_{5}T_{7}}{\rho^{2}N^{2}T_{4}},\;\;\;T_{7}=\exp\left(-36\frac{\rho^{2}N^{2}T_{3}}{T_{2}}t\right). \end{array}$$

Finally,

(71) $$\begin{array}{c} R_{n}\left(t\right)=1+{\left(T_{3}/T_{6}\right)}^{1/2}T_{2}T_{4}T_{6}^{2}[-26244\frac{\beta_{3}\tau^{2}T_{3}^{6}T_{5}^{2}T_{7}^{2}}{\rho T_{2}^{2}T_{4}^{2}T_{6}^{4}{\left(T_{3}/T_{6}\right)}^{3/2}}+104976\frac{\beta_{3}\tau^{2}T_{3}^{5}T_{5}^{2}T_{7}^{2}}{\rho T_{2}^{2}T_{4}^{2}T_{6}^{3}{\left(T_{3}/T_{6}\right)}^{1/2}}-\\ 5832\frac{\beta_{3}\rho \tau N^{2}T_{3}^{4}T_{5}T_{7}}{T_{2}^{2}T_{4}T_{6}^{2}{\left(T_{3}/T_{6}\right)}^{1/2}}]/\left(162\beta_{3}\tau T_{3}^{3}T_{5}T_{7}\right). \end{array}$$

The above results are valid if ${\left(\frac{\tau R}{\rho N}\right)}^{3}<<1$. This means that

(72) $$\begin{array}{c} \frac{\tau^{3}}{\rho^{3}N^{3}}\{\frac{\rho N}{3\tau }+\beta_{3}\{T3/[[-\frac{T_{2}}{18\tau \rho N}+9\tau T_{3}(7\tau \beta_{3}^{2}\rho^{2}N^{2}+27\tau^{4}\beta_{3}^{2}-\tau \beta_{3}^{2}T_{1}^{1/2}-81\tau^{3}\rho \beta_{3}^{2}+\\ 9\rho^{3}N^{3})\exp\left(-36\frac{\rho^{2}N^{2}T_{3}}{T_{2}}t\right)]/\left(\rho^{2}N^{2}\left(7\rho^{2}N^{2}+27\tau^{3}-T_{1}^{1/2}-81\rho \tau^{2}\right)\right)]{\}}^{1/2}{\}}^{3}<<1 \end{array}$$

There are other solutions of this kind. They require specific values of one or several parameters connected to the epidemic wave. These specific values required decrease the probability of realization of the corresponding wave. Because of this, we will discuss this kind of specific solution elsewhere.

Next, we briefly discuss the exact solutions obtained, which are not appropriate for the use of the purposes of the SIR model. These solutions are (30), (39), and (44). The problems are connected to the values of the parameters $\alpha_{i}$ corresponding to the SIR model. Let us consider the solution (30). From (28), we have the requirement $\alpha_{0}=\frac{\alpha_{2}^{3}}{27\alpha_{3}^{2}}$. However, from (13), it follows that $\alpha_{2}<0$ and $\alpha_{3}>0$. Thus, we have $\alpha_{0}=\rho \left[N-S\left(0\right)\right]<0$ and then N<S(0). The last relationship is false from the point of view of the SIR model. Thus, (30) is a valid solution, but it cannot be used for the purposes of the SIR model.

The next solution is (39). Here, we have the same problem with the parameter $\alpha_{0}$, which has to be positive. However, $\alpha_{0}=\alpha_{3}^{4}/\left(256\alpha_{4}^{3}\right)$ and $\alpha_{3}>0$, $\alpha_{4}<0$ for the specific case of the SIR model. Thus, we can not use (39) to model epidemic waves within the SIR model.

Next, we consider solution (44). Here, we have the same problem with $\alpha_{0}$, $\alpha_{4}$ and $\alpha_{5}$ as for the last two solutions for the specific case of the parameters of the SIR model. Therefore, we cannot use (44) to model epidemic waves within the SIR model.

## 5. Epidemic Waves Based on Some of the Obtained Solutions

Let us consider the influence of the parameters of the SIR model on the spread of the epidemic wave. The study will be made on the basis of some of the solutions obtained above.

**Figure 1:**
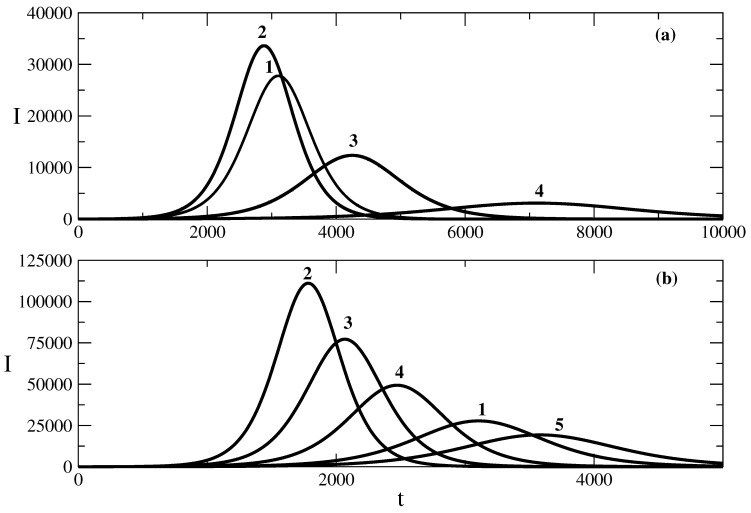
Influence of the recovery rate ρ on the number of infected people. Figure (**a**): the solution (50). Curve 1: basic solution with parameters $N=10^{6}$, S(0) = 999,990, τ=0.009, ρ=0.006. For the other curves, there are changes only in the value of the parameter ρ. Curve 2: ρ=0.0057, Curve 3: ρ=0.007, Curve 4: ρ=0.008. Figure (**b**): the solution (58). Curve 1: basic solution with parameters $N=10^{6}$, S(0) = 999,990, τ=0.009, ρ=0.006, $D=10^{5}$, E=1. For the other curves, there are changes only in the value of the parameter ρ. Curve 2: ρ=0.003 = Curve 3: ρ=0.004. Curve 4: ρ=0.005. Curve 5: ρ=0.0065.

Figure 1 shows the influence of the recovery rate ρ of the SIR model on the shape of the epidemic wave for the case of the relationship (50) obtained on the basis of solution (49). The decrease in the recovery rate leads to a larger peak of the wave (larger value of the maximum number of infected individuals for the studied wave). In addition, the peak of the wave occurs earlier. The increase in the value of the recovery rate ρ leads to a decrease in the maximum number of infected individuals. In addition, the peak of the epidemic wave is postponed, as can be seen from curves 3 and 4 in Figure 1a. The same kind of dependence on the maximum and the shape of the epidemic wave on the recovery rate ρ is observed for the relationship (58) for the epidemic wave obtained on the basis of solution (57) to the equation connected to the SIR model. Thus, the influence of the recovery rate on the epidemic wave is that the increased recovery rate leads to a faster decrease in the number of infected individuals, and this slows the rise of the epidemic wave and decreases its height.

**Figure 2:**
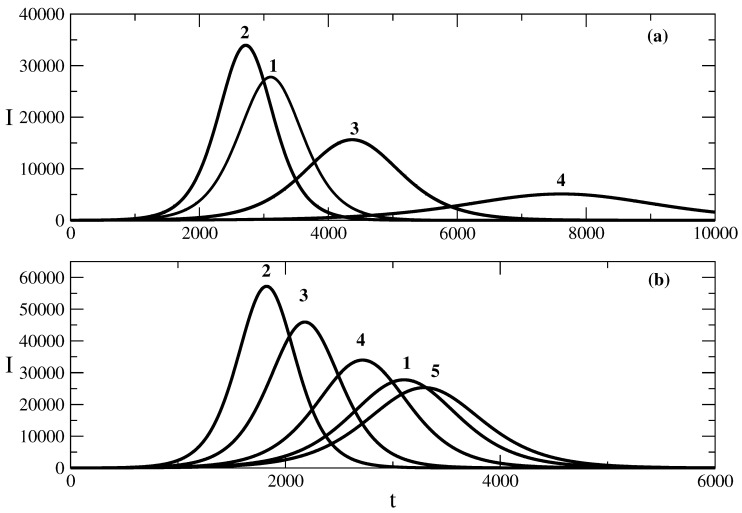
Influence of the transmission rate τ on the number of infected people. Figure (**a**): the solution (50). Curve 1: basic solution with parameters $N=10^{6}$, S(0) = 999,990, τ=0.009, ρ=0.006. For the other curves, there are changes only in the value of the parameter τ. Curve 2: τ=0.0095, Curve 3: τ=0.008, Curve 4: τ=0.007. Figure (**b**): the solution (58). Curve 1: basic solution with parameters $N=10^{6}$, S(0) = 999,990, τ=0.009, ρ=0.006, $D=10^{5}$, E=1. For the other curves, there are changes only in the value of the parameter τ. Curve 2: τ=0.0115. Curve 3: τ=0.0105. Curve 4: ρ=0.0095. Curve 5: ρ=0.0088.

Figure 2 shows the influence of the transmission rate τ on the shape of the epidemic wave. The increase in the transmission rate for the case of relationship (50) obtained by solution (49) leads to an increase in the value of the maximum number of infected individuals for the wave. In addition, the wave rises faster, as can be seen from curves 1 and 2 of Figure 2a. The effect of the decrease in the transmission rate on the shape of the wave described by the relationship (58) obtained by solution (57) is shown in Figure 2b. The decrease in τ, in this case, leads to a smaller maximum of the epidemic wave, and the wave occurs later. Thus, the increase in the transmission rate leads to a faster occurrence of the epidemic wave and an increase in the maximum number of infected individuals for the corresponding epidemic wave.

**Figure 3:**
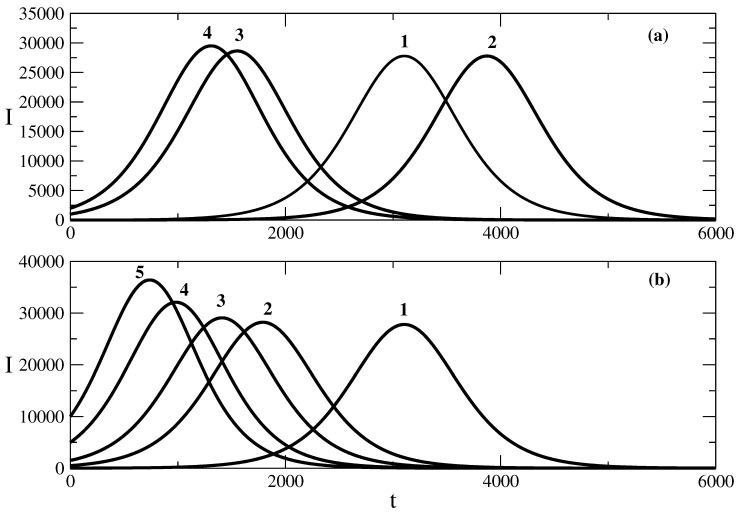
Influence of the parameter S(0) on the number of infected people. Figure (**a**): the solution (50). Curve 1: basic solution with parameters $N=10^{6}$, S(0) = 999,990, τ=0.009, ρ=0.006. For the other curves, there are only changes in the value of parameter S(0). Curve 2: S(0) = 999,999, Curve 3: S(0) = 999,000, Curve 4: S(0) = 998,000. Figure (**b**): the solution (58). Curve 1: basic solution with parameters $N=10^{6}$, S(0) = 999,990, τ=0.009, ρ=0.006, $D=10^{5}$, E=1. For the other curves, there are changes only in the value of parameter S(0). Curve 2: S(0) = 999,500. Curve 3: S(0) = 998,500. Curve 4: S(0) = 995,000. Curve 5: S(0) = 990,000.

Figure 3 shows the influence of the initial number S(0) of susceptible individuals on the shape of the epidemic wave. We note that at t=0, we assume R(0)=0 and then N=S(0)+I(0). Then, the decrease in S(0) means that we have a larger value of I(0). In other words, the decrease in the initial number of susceptible individuals means that the epidemic wave starts with a larger initial number of infected individuals. The influence of S(0) on the shape of the wave described by (50) obtained on the basis of solution (49) is shown in Figure 3a. The decrease in the initial number of susceptible people (the increase in the initial number of infected individuals) leads to a faster rise of the epidemic wave and a larger value of the maximum number of infected individuals. The result of the influence of S(0) on the epidemic wave described by relationship (58) (obtained on the basis of solution (57)) is the same, as can be seen in Figure 3b. Then, the larger value of suspected individuals at t=0 (the smaller cluster of infected individuals at t=0) leads to a later occurrence of the epidemic wave and a decrease in its height.

The above results of the influence of the parameters ρ, τ and S(0) on the shape of the epidemic wave hint at a strategy for fighting the epidemic. One needs to detect the epidemic when the cluster of infected individuals is still small. Then one has to try to decrease the transmission rate and increase the recovery rate. This can lead to a later occurrence of the epidemic wave and a decrease in the height of this wave.

**Figure 4:**
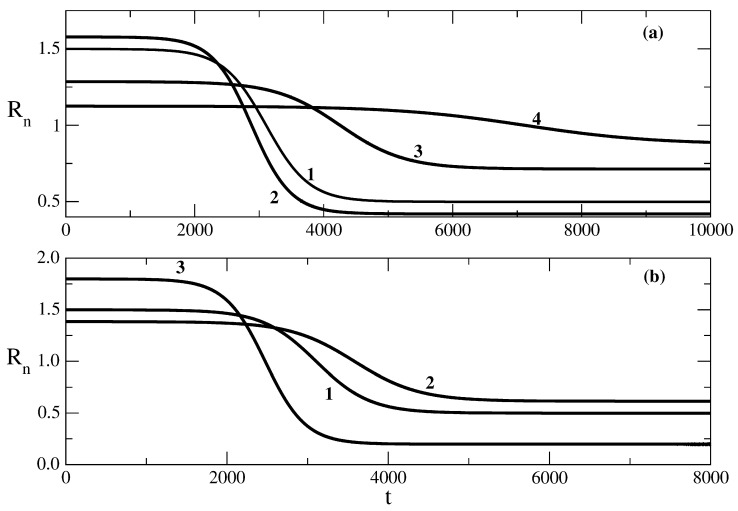
Influence of the recovery rate ρ on the effective reproduction number $R_{n}$ from Equation (47). Figure (**a**): the relationship (53) obtained on the basis of the solution (49). Curve 1: basic solution with parameters $N=10^{6}$, S(0) = 999,990, τ=0.009, ρ=0.006. For the other curves, there are changes only in the value of the parameter ρ. Curve 2: ρ=0.0057, Curve 3: ρ=0.007, Curve 4: ρ=0.008. Figure (**b**): the relationship (62) obtained on the basis of the solution (58). Curve 1: basic solution with parameters $N=10^{6}$, S(0) = 999,990, τ=0.009, ρ=0.006, $D=10^{5}$, E=1. For the other curves, there are only changes in the value of the parameter ρ. Curve 2: ρ=0.0065. Curve 3: ρ=0.005.

The following figures show the influence of the parameters of the SIR model on the effective reproduction number $R_{n}$ connected to the epidemic wave. In principle, at the beginning of the wave, $R_{n}$ is larger than 1, and at the end of the wave, $R_{n}$ is smaller than 1. Figure 4 shows the influence of the recovery rate ρ on the effective reproduction number $R_{n}$. Figure 4a shows the situation for the case of relationship (53) obtained on the basis of solution (49). We see that the decrease in the value of ρ leads to an increase in the initial value of $R_{n}$, which is followed by a large decrease in the value of the effective reproduction number in the course of the value (see curves 1 and 2 of Figure 4a). The increase in the value of ρ results in a smaller initial value of $R_{n}$ and a smaller decrease in its value in the course of a wave. Large enough values of ρ lead to values of $R_{n}$, which are close to 1 and correspond to a slowly rising epidemic wave.

Figure 4b shows the situation for the relationship (62) obtained on the basis of the solution (58). Quantitatively, the situation is the same as above. The increase in the value of the recovery rate leads to a decrease in the initial value of the effective reproduction number and to a smaller decrease in the value of this number in the course of the wave.

**Figure 5:**
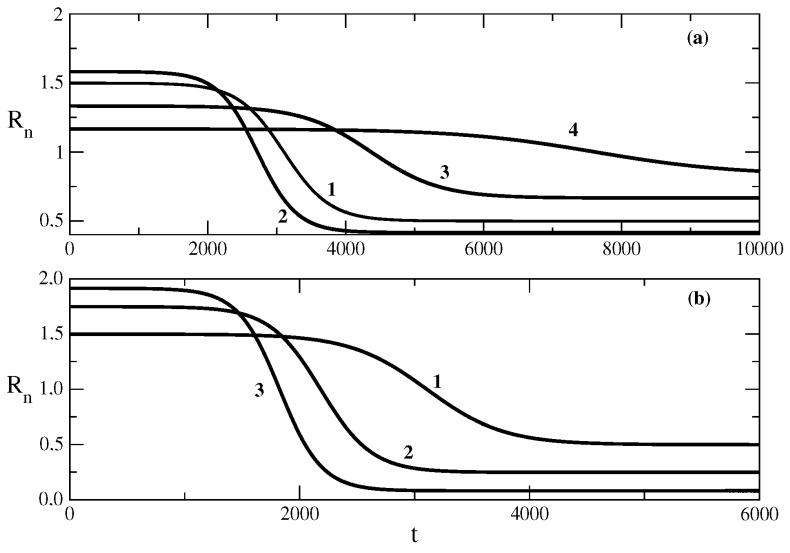
Influence of the transmission rate τ on the effective reproduction number $R_{n}$ Equation (47). Figure (**a**): the relationship (53). Curve 1: basic solution with parameters $N=10^{6}$, S(0) = 999,990, τ=0.009, ρ=0.006. For the other curves, there are changes only in the value of the parameter τ. Curve 2: τ=0.0095, Curve 3: τ=0.008, Curve 4: τ=0.007. Figure (**b**): the relationship (62). Curve 1: basic solution with parameters $N=10^{6}$, S(0) = 999,990, τ=0.009, ρ=0.006, $D=10^{5}$, E=1. For the other cures, there are changes only in the value of the parameter τ. Curve 2: τ=0.0105. Curve 3: τ=0.0115.

Figure 5 shows the influence of the transmission rate τ on the evolution of the effective reproduction number $R_{n}$ in the course of an epidemic wave. Figure 5a shows the situation for the case of relationship (53) obtained on the basis of solution (49). In this case, the decrease in the transmission rate leads to a decrease in the initial value of the effective reproduction number $R_{n}$ and to a smaller interval of decrease in the value of $R_{n}$ in the course of the epidemic wave. The same situation can be observed in Figure 5b for the case of relationship (62) obtained on the basis of the solution (58). We note that an appropriate value of the transmission rate combined with the corresponding values of the other parameters can make the value of $R_{n}$ closer to 2 and become even larger than this value.

**Figure 6:**
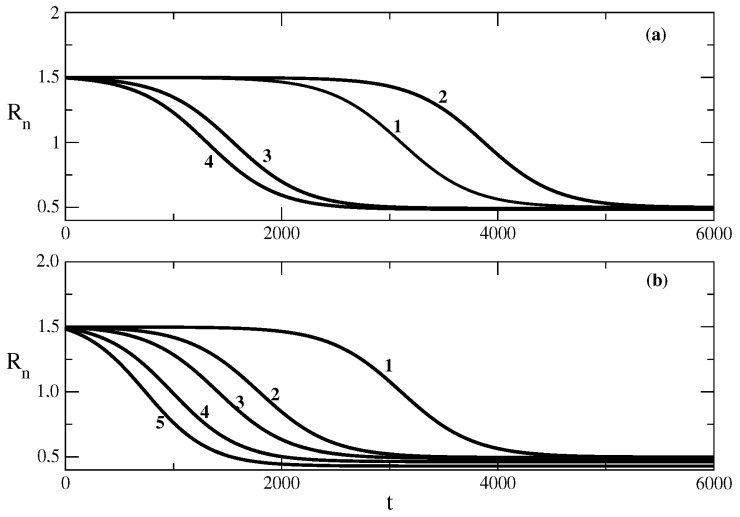
Influence of the parameter S(0) on the effective reproduction number $R_{n}$ Equation (47). Figure (**a**): the relationship (53). Curve 1: basic solution with parameters $N=10^{6}$, S(0) = 999,990, τ=0.009, ρ=0.006. For the other curves, there are changes only in the value of parameter S(0). Curve 2: S(0) = 999,999, Curve 3: S(0) = 999,000, Curve 4: S(0) = 998,000. Figure (**b**): the relationship (62). Curve 1: basic solution with parameters $N=10^{6}$, S(0) = 999,990, τ=0.009, ρ=0.006, $D=10^{5}$, E=1. For the other curves, there are changes only in the value of the parameter S(0). Curve 2: S(0) = 999,500. Curve 3: S(0) = 998,500. Curve 4: S(0) = 995,000. Curve 5: S(0) = 990,000.

Figure 6 shows the influence of the initial number of susceptible individuals S(0) on the value of the effective reproduction number $R_{n}$. Figure 6a shows the situation for the case of relationship (53) obtained on the basis of solution (49). The decrease in the initial number of susceptible individuals (which corresponds to a larger number of infected individuals at t=0) leads to a faster decrease in the value of the effective reproduction number $R_{n}$. The same result can be seen in Figure 6b for relationship (62) obtained on the basis of solution (58).

**Figure 7:**
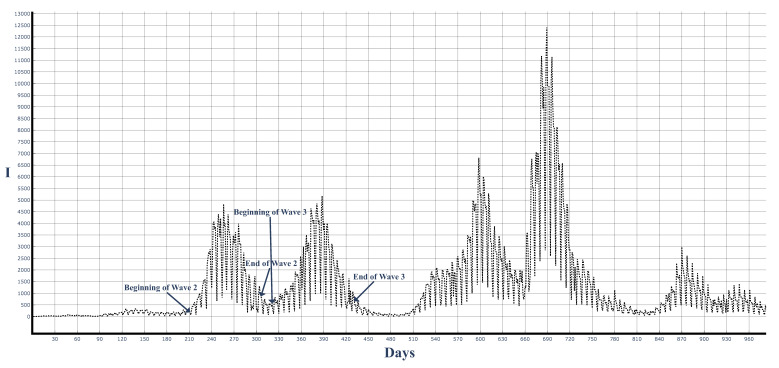
COVID-19 epidemic waves in Bulgaria. X-axis: days since the beginning of the pandemic in Bulgaria (8-th of March 2020). Y-axis: registered number *I* of infected people per day. Wave 2 and wave 3 will be compared to the analytical results in this article.

Finally, we will use solutions (50) and (58) to approximate real data from the COVID-19 pandemic in Bulgaria. The data for the infected individuals for the first approximately 1000 days of the pandemic are shown in Figure 7. There have been several large COVID-19 epidemic waves in Bulgaria (the population of which is approximately 6.8 million people). In this article, we show how the above analytic results can be related to the second and third COVID-19 waves.

Figure 7 shows that there are periodic drops in the number of cases on Saturdays and Sundays and increases in the number of cases on Mondays. In order to remove this effect, which exists because of the presence of holidays, below we will work with the 7-day averages of the data $I_{i}^{*}=\frac{1}{7}\sum_{j=i-3}^{i+3}I_{j}$ and with the 14-day average of the data: $I_{i}^{*}=\frac{1}{14}\sum_{j=i-6}^{i+7}I_{j}$.

**Figure 8:**
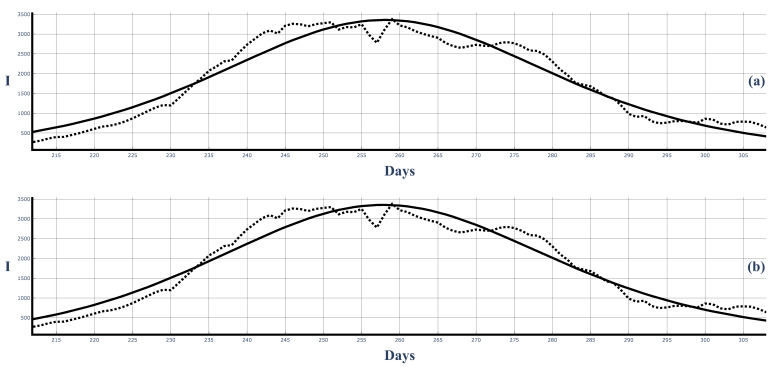
The second large COVID-19 wave in Bulgaria and the best fit of the 7-day-averaged data with the solutions (50) and (58). Dots: infected people (7-day average). Solid curves: Figure (**a**): solution (50). ρ=0.0000982, τ=0.007878. Figure (**b**): solution (58). ρ=0.0000893, τ=0.007883.

Figure 8 shows the second COVID-19 wave in Bulgaria (dotted line shows the 7-day-average data) and its best fit with solutions (50) (Figure 8a) and (58) (Figure 8b). We observe that the fit with solution (58) is better, especially in the beginning and end regions of the wave. This better fit is also observed in the other figures below.

**Figure 9:**
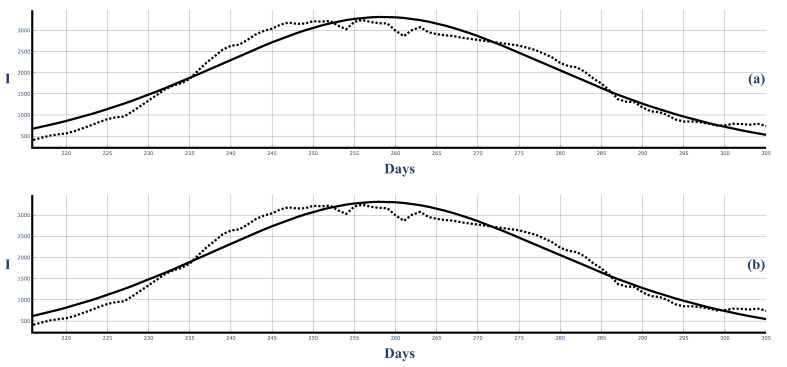
The second large COVID-19 wave in Bulgaria and the best fit of the 14-day-average data from the solutions (50) and (58). Dots: infected people (14-day average). Solid curves: Figure (**a**): solution (50). ρ=0.0000985, τ=0.007875. Figure (**b**): solutions (58). ρ=0.0000813, τ=0.007863.

Figure 9 shows the fit of the 14-day averages of the data from solutions (50) and (58). Again, the fit by (58) is better. This can be expected as (58) is a more general solution in comparison to (50). The 14-day data are smoother than the 7-day-average data, and because of this, the fit of the 14-day-average data is better that the fit of the 7-day-average data.

**Figure 10:**
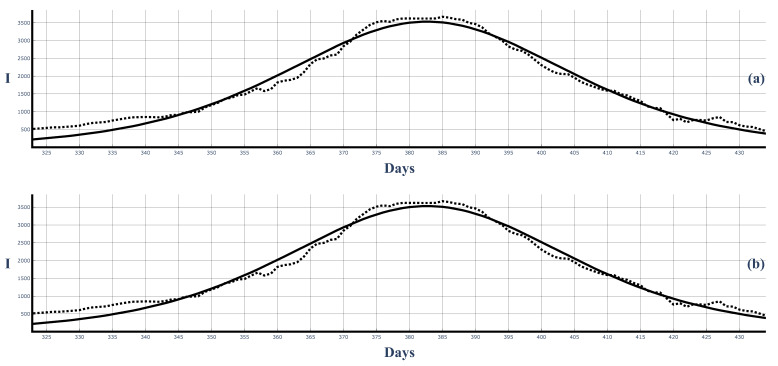
The third large COVID-19 wave in Bulgaria and the best fit of the 7-day-averaged data by the solutions (50) and (58). Dots: infected people (7-day average). Solid curves: Figure (**a**): solution (50). ρ=0.0000598, τ=0.005285. Figure (**b**): solutions (58). ρ=0.0000599, τ=0.005296.

**Figure 11:**
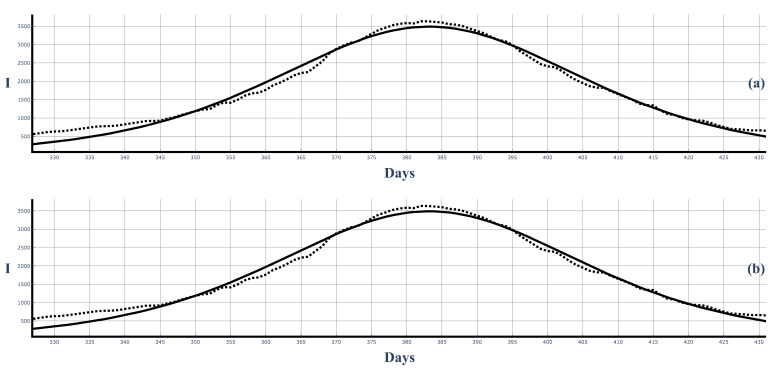
The third large COVID-19 wave in Bulgaria and the best fit of the 14-day-averaged data from solutions (50) and (58). Dots: infected people 14-day average). Solid curves: Figure (**a**): solution (50). ρ=0.0000676, τ=0.005326. Figure (**b**): solutions (58). ρ=0.0000699, τ=0.005299.

Figure 10 and Figure 11 show the third large COVID-19 wave in Bulgaria and the corresponding fits of the 7-day-average data and 14-day-average data.

On the basis of the COVID-19 data and their fits, we can obtain the parameters of the models and compare these parameters for the two studied COVID-19 waves in Bulgaria. The comparison of the values of ρ (the recovery rate) obtained by the fits of the data for COVID-19 spreading in Bulgaria shows that ρ was larger for the second large wave in comparison to the third large wave. In addition, the transmission rate τ for the second large wave was larger in comparison to the transmission rate for the third large wave. Thus, the result is that the version of the COVID-19 virus that was responsible for the second large COVID- wave in Bulgaria spread faster than the version of the virus that was responsible for the third wave. In addition, the recovery time of the second large wave was faster in comparison to the recovery time of the third large wave.

## 6. Concluding Remarks

In this article, we apply the Simple Equations method (SEsM) to a chain of nonlinear differential equations connected to the SIR model of epidemic spreading. We obtain three classes of solutions from the point of their applicability for the purpose of epidemic modeling. The first class of solutions can be applied to model epidemics without imposing additional restrictions containing equalities among the parameters of the SIR model. Such solutions are (22) and (25). The second class of obtained solutions is solutions that require additional relationships containing equalities among the parameters of the SIR model. Such a solution is (34). The third class of the obtained solutions is solutions to the corresponding equation of the chain of equations but solutions that can not be used for the purpose of modeling epidemic spread. We note that the obtained solutions are appropriate for the description of a single epidemic wave that affects some populations and leads to an infection of a relatively small percentage of the individuals of this population. The obtained analytical solution allows us to study the influence of the parameters of the model (such as transmission rate, recovery rate and initial number of susceptible individuals) on the shape and evolution of the epidemic wave. The results are that larger recovery rates, smaller transmission rates and a larger number of potentially affected individuals (small number of infected individuals at the beginning of the wave) lead to a slower advancement of the wave and a decrease in its amplitude.

The obtained solution from the second class of solutions is also quite interesting. However, for its practical realization, it is required that specific relationships among the parameters of the epidemics are presented. There is more than one solution in this class of solutions to the chain of the studied equations. We intend to study these solutions elsewhere.

Finally, the third class of solutions demonstrates the capacity of SEsM. The methodology leads to numerous exact solutions to various equations, and this has been demonstrated many times already. The obtained solutions of this class can not be used to model epidemic waves as they lead to some relationships among the parameters of the SIR model that correspond to unrealistic assumptions about these parameters. Nevertheless, the obtained solutions are solutions to the corresponding equations from the studied chain of equations and can be used in other models where the corresponding relationships among the parameters lead to acceptable assumptions about the model parameters.

## Data Availability

Data for COVID-19 registered cases in Bulgaria can be found at https://coronavirus.bg/.
